# MDM2 promotes genome instability by ubiquitinating the transcription factor HBP1

**DOI:** 10.1038/s41388-019-0761-2

**Published:** 2019-02-28

**Authors:** Zhengyi Cao, Junhui Xue, Yuning Cheng, Jiyin Wang, Yujuan Liu, Hui Li, Wei Jiang, Gang Li, Yaoting Gui, Xiaowei Zhang

**Affiliations:** 1 Department of Biochemistry and Molecular Biology, School of Basic Medical Sciences, Beijing Key Laboratory of Protein Posttranslational Modifications and Cell Function, Peking University Health Science Center, Beijing, 100191 P. R. China; 2 Guangdong and Shenzhen Key Laboratory of Male Reproductive Medicine and Genetics, Institute of Urology, Peking University Shenzhen Hospital, Shenzhen-Peking University-the Hong Kong University of Science and Technology Medical Center, Shenzhen, 518000 P. R. China

**Keywords:** DNA damage and repair, Biochemistry

## Abstract

Genome instability is a common feature of tumor cells, and the persistent presence of genome instability is a potential mechanism of tumorigenesis. The E3 ubiquitin ligase MDM2 is intimately involved in genome instability, but its mechanisms are unclear. Our data demonstrated that the transcription factor HBP1 is a target of MDM2. MDM2 facilitates HBP1 proteasomal degradation by ubiquitinating HBP1, regardless of p53 status, thus attenuating the transcriptional inhibition of HBP1 in the expression of its target genes, such as the DNA methyltransferase DNMT1 and histone methyltransferase EZH2, which results in global DNA hypermethylation and histone hypermethylation and ultimately genome instability. The repression of HBP1 by MDM2 finally promotes cell growth and tumorigenesis. Next, we thoroughly explored the regulatory mechanism of the MDM2/HBP1 axis in DNA damage repair following ionizing radiation. Our data indicated that MDM2 overexpression-mediated repression of HBP1 delays DNA damage repair and causes cell death in a p53-independent manner. This investigation elucidated the mechanism of how MDM2 promotes genome instability and enhances tumorigenesis in the absence of p53, thus providing a theoretical and experimental basis for targeting MDM2 as a cancer therapy.

## Introduction

Mouse double minute 2 (*MDM2*) is an oncogene that promotes cell growth, survival, invasion, and therapeutic resistance [1]. MDM2 is a protein with multiple functions, of which E3 ubiquitin ligase is the most widely studied function. The tumor suppressor p53 is antagonized by MDM2 under physiological conditions [2]. MDM2 reduces the stability of p53 by ubiquitinating it for proteasomal degradation. Furthermore, p53-mediated transactivation is inhibited and the subcellular localization of p53 is affected by MDM2. In several human and murine malignancies, MDM2 is frequently observed overexpression [3–5], and studies suggest that changes in MDM2 expression break the balance p53 signaling, which ultimately influences tumorigenesis [6–8].

While the primary function of MDM2 is to regulate p53, there is evidence that MDM2 regulates tumorigenesis in a p53-independent way [9]. Overexpression of MDM2 in *p53*-null mice, the increasing incidence of sarcoma is observed compare with *p53*-null mice alone [10]. This finding suggests that overexpression of MDM2 is capable of promoting tumorigenesis and transforming cells in vitro, independent of p53 signaling pathway [11]. The high rate of MDM2 amplification is consistent in human and mice sarcomas [12, 13].

As an E3 ubiquitin ligase, MDM2 also promotes ubiquitination of itself, its homolog MDM4 (also known as MDMX) [14], and several other cellular proteins, including androgen receptor, Tip60, FoxO3A, E-cadherin, and Slug [15–19]. In fact, many studies have shown that MDM2 is also involved in the regulation of a number of cell cycle regulation and apoptosis-related proteins, such as p21, RASSF6, RNF2, and p73 [8, 20–23]. These studies suggest that MDM2 also has other functions in addition to merely regulating p53.

Recently, increasing evidence has indicated that overexpression of MDM2 can induce genome instability in the absence of p53. For example, increased ploidy is a marker of genome instability. The increased ploidy of mammary epithelial cells is not associated with p53 expression in mammary-specific *MDM2*-trasngenic mice [24]. Nbs1 is a member of the Mre11/Ras50/Nbs1 (M-R-N) complex that is responsible for DNA damage repair. MDM2 interacts with Nbs1 and inhibits the function of the M-R-N complex, which induced chromosome breakage and delayed DNA damage repair [25]. Furthermore, Wienken et al. identified an important role for MDM2 in maintaining stemness and cancer cell survival via stabilizing histone modifications, such as H2AK119ub1 and H3K27me3 [26]. In this study, we found an unexpected connection between MDM2 and the transcription factor HBP1, which our previous studies have shown to regulate DNA and histone methylation, and ultimately genome stability.

Transcription factors HBP1 is a member of the sequence-specific high-mobility-group (HMG) family. As a transcription factor, HBP1 represses its target genes at the transcriptional level, such as *p47phox*, *N-MYC*, *C-MYC*, *DNMT1*, and *EZH2*, via directly combined with the high-affinity elements [27–31]. Additionally, HBP1 also plays a transcriptional activation role on several genes, including those encoding p21, p16, myeloperoxidase (MPO), and histone H1 [31–35]. The differences in DNA binding elements, degree of HBP1 acetylation, promoter sequences and histone modification determine the repression or activation transcriptional activities of HBP1 on different downstream genes. Given that HBP1 targets a number of important cell cycle regulators, overexpression of HBP1 arrested cell cycle and inhibited tumorigenesis in various cells types and organs is conceivable.

Genome instability is a common feature associated with almost all human cancers, but the role of MDM2 in promoting genome instability independent of p53 requires further investigation. In this study, we explored the effects of MDM2 on HBP1 ubiquitination and found an intricate posttranslational modification mechanism that is involved in regulating genome instability. We identified that MDM2 decreased HBP1 protein stability and transcriptional activity by interacting with HBP1 and promoting HBP1 ubiquitination. The net results of MDM2 repression of HBP1 were genome instability and tumorigenesis, which were attributed to enhanced expressions of the DNA methyltransferase *DNMT1* and the histone methyltransferase *EZH2*, which are transcriptional repression targets of HBP1. Additionally, we thoroughly explored the regulatory mechanism of the MDM2/HBP1 signaling pathway on DNA break repair following ionizing radiation. Blocking the repression of HBP1 by MDM2 facilitated DNA repair processes after ionizing radiation-induced DNA damage, whereas MDM2 overexpression-mediated HBP1 repression delayed DNA break repair and caused cell death in a p53-independent manner. Together, the MDM2/HBP1 axis constitutes a novel regulatory node that induces genome instability and tumorigenesis. Thus, the balance of MDM2 and HBP1 is critical for maintaining genome stability and proper cellular metabolism following ionizing radiation.

## Results

### MDM2 reduced HBP1 protein expression

We have previously reported that HBP1 enhances p53 stability by inserting into the p53-MDM2 complex and inhibiting MDM2-mediated p53 ubiquitination [31]. As an E3 ubiquitin ligase, MDM2 binds to critical signal transduction molecules, such as p53, E-cadherin and Chk2, ubiquitinating them for proteasomal degradation [36]. Thus, we explored whether HBP1 is another target of MDM2 and whether MDM2 promotes genome instability by ubiquitinating HBP1, as HBP1 represses the transcription of *DNMT1* and *EZH2*, which are involved in genome stability.

**Fig. 1:**
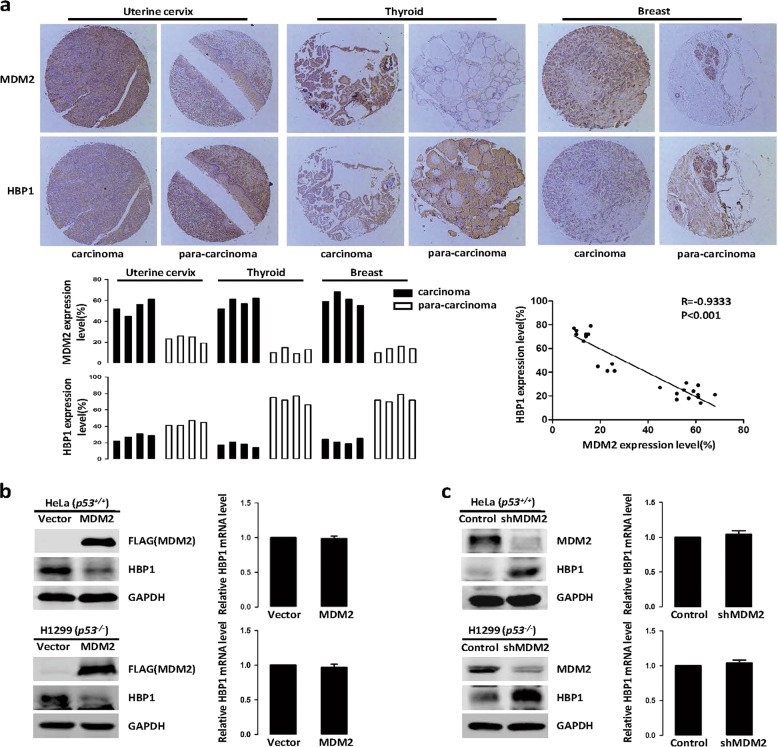
MDM2 reduces HBP1 protein expression. **a** There is an inverse correlation between MDM2 and HBP1 expression in some types of human cancer with high expression of MDM2. Tissue microarray slides were stained with MDM2 or HBP1 antibodies, followed by secondary antibodies labeled biotin, and colored by streptavidin ALP system (top panel). Stained areas were quantitated with Image J software (bottom, left panel), the linear regression and correlation were analyzed by GraphPad Prism 5 (bottom, right panel). **b** MDM2 overexpression decreases HBP1 protein expression. The protein levels of MDM2 and HBP1 were measured by western blotting in HeLa (*p53*^*+/+*^) and H1299 (*p53*^*−/−*^) cells transfected with PRK5-MDM2 or PRK5 (as a control). GAPDH was used as a loading control (left panel). The mRNA level of HBP1 was measured by Real-time PCR in HeLa and H1299 cells transfected with PRK5-MDM2 or PRK5 (right panel). **c** MDM2 knockdown by shRNA increases HBP1 protein expression. The protein levels of MDM2 and HBP1 were measured by western blotting in HeLa and H1299 cells stably transfected with pLL3.7-shMDM2 or pLL3.7 (as a control) through lentiviral infection. GAPDH was used as a loading control (left panel). The mRNA level of HBP1 was measured by real-time PCR in HeLa and H1299 cells stably transfected with pLL3.7-shMDM2 or pLL3.7 (right panel) through lentiviral infection

Intriguingly, we observed a statistically significant inverse correlation between MDM2 and HBP1 protein levels in human uterine cervix cancer, thyroid cancer, and breast cancer with high expression of MDM2 (compared with carcinoma versus para-carcinoma) using immunohistochemistry assay (Fig. 1a). To determine whether MDM2 represses HBP1 expression, we exogenously overexpressed MDM2 in HeLa (*p53*^*+/+*^) and H1299 (*p53*^*−/−*^) cells. Exogenous MDM2 expression decreased HBP1 protein levels in both HeLa and H1299 cells (left panel) but had no effect on the mRNA levels of HBP1 (right panel) in Fig. 1b. To address the functions of endogenous MDM2 and HBP1, we used short hairpin RNAs (shRNAs) to knockdown MDM2 expression (Fig. 1c). MDM2 knockdown increased HBP1 protein levels (left panel) in these two cell lines but had no effect on the mRNA levels of HBP1 (right panel). Therefore, MDM2 clearly inhibited HBP1 protein expression at a posttranscriptional level. Furthermore, the effect of MDM2 on HBP1 protein expression was p53 independent.

### MDM2 inhibited HBP1 protein expression by reducing its stability

**Fig. 2:**
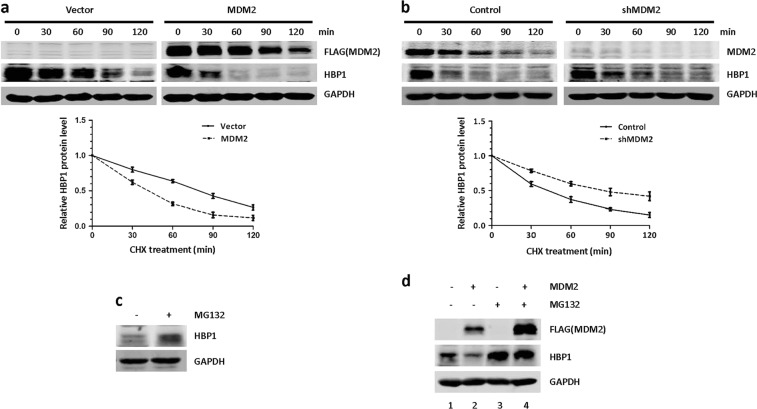
MDM2 inhibits HBP1 protein expression by reducing its stability. **a**, **b** MDM2 shortens the half-life of HBP1. HeLa cells were transfected with PRK5-MDM2, PRK5 **a** or stably transfected with pLL3.7, pLL3.7-shMDM2 **b** through lentiviral infection, respectively. Cells were incubated with the protein synthesis inhibitor cycloheximide (CHX) for 0, 30, 60, 90, or 120 min before collect. HBP1 and MDM2 protein levels were detected by western blotting. GAPDH was used as the loading control for this turnover experiment (top panel). Quantification of HBP1 protein levels was determined using Image J software normalized to GAPDH and densitometry was plotted for the average ± S.D. of three-independent experiments (bottom panel). **c** HBP1 protein level is elevated in the presence of MG132. HeLa cells were treated with MG132 for 6 h, the protein level of HBP1 was measured by western blotting. GAPDH was used as a loading control. **d** MDM2 does not decrease HBP1 protein level in the presence of MG132. HeLa cells were transfected with PRK5-MDM2 or PRK5. Forty-two hours after transfection, cells were incubated with (lanes 3 and 4) or without (lanes 1 and 2) MG132 for another 6 h. HBP1 protein was detected by western blotting. GAPDH was used as a loading control

We next investigated whether MDM2 decreased HBP1 protein levels by reducing the stability of HBP1. As shown in Fig. 2a, b, MDM2 overexpression obviously reduced HBP1 stability, whereas knocking down MDM2 by shRNA distinctly increased HBP1 stability. Furthermore, the HBP1 protein level was increased by the proteasome inhibitor MG132, and MDM2 overexpression did not depress HBP1 when the proteasome was inhibited by MG132 (Fig. 2c, d), which suggested that MDM2 downregulates HBP1 protein levels in a proteasome-dependent manner. These results indicated that MDM2 inhibits HBP1 expression by decreasing its stability.

### MDM2 interacted with HBP1 in vivo and in vitro

**Fig. 3:**
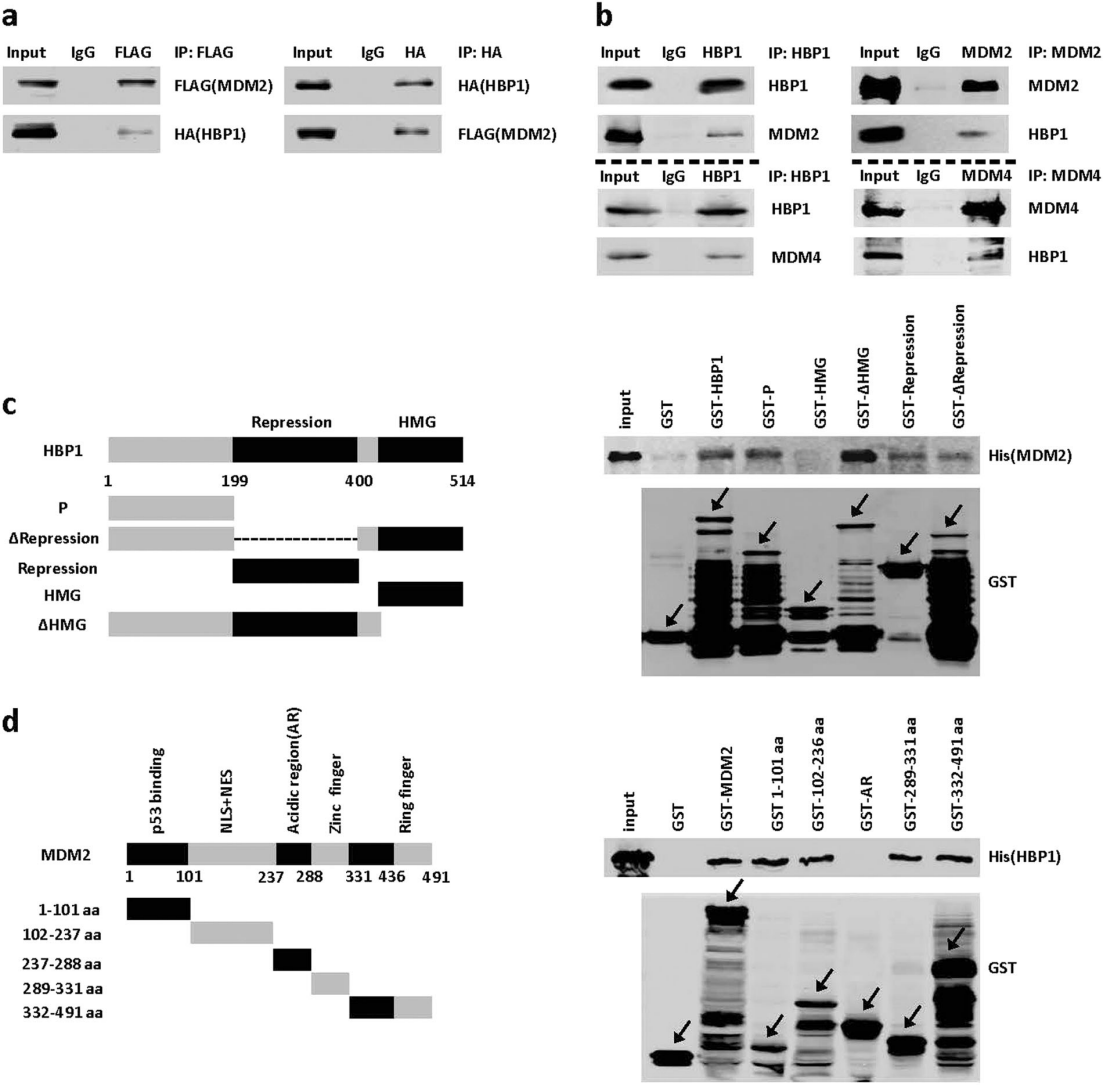
MDM2 interacts with HBP1 in vivo and in vitro. **a**, **b** MDM2 interacts with HBP1 in vivo. 293T cells were co-transfected with HA-HBP1 and FLAG-MDM2. IP assay was carried out by using anti-FLAG/HA antibody and followed by western blotting with anti-HA/FLAG antibody. The same samples were immunoblotted against FLAG/HA to determine immunoprecipitation efficiency **a**. H1299 cells were lysed with IP lysis buffer and then subjected to immunoprecipitation with anti-HBP1, anti-MDM2 or anti-MDM4, and MDM2, MDM4 or HBP1 antibody followed by western blotting analysis **b**. **c**, **d** MDM2 interacts with HBP1 in vitro. Schematic representation of N-terminal GST-tagged full-length HBP1 along with its various deletion mutants (**c**, left panel), MDM2 along with its various deletion mutants (**d**, left panel). GST pulldown assay was carried out to determine the domain of HBP1 essential for its interaction with MDM2 (**c**, right panel), and the domain of MDM2 essential for its interaction with HBP1 (**d**, right panel). GST pulldown efficiency was evaluated by western blotting with anti-GST antibody

We next sought to clarify the underlying mechanism by which MDM2 decreases the stability of HBP1. We hypothesized that HBP1 may be a target gene of MDM2, MDM2 may regulate HBP1 by physically interacting with HBP1. First, we co-transfected FLAG-tagged MDM2 and HA-tagged HBP1 into 293T cells. Twenty-four hours after transfection, co-immunoprecipitation assays were carried out with anti-FLAG or anti-HA antibodies, followed by western blotting analysis. As shown in Fig. 3a, exogenous MDM2 interacted with exogenous HBP1 in 293T cells. Then we carried out co-immunoprecipitation experiments in *p53*-null H1299 cells with anti-MDM2 or anti-HBP1 antibodies, and analyzed by western blotting. As shown in Fig. 3b, endogenous MDM2 also interacted with endogenous HBP1 in H1299 cells. In addition, we also verified that HBP1 binds to MDM2/MDM4 complex, as MDM4 is a homolog of MDM2 which shares similar structure and heterodimerizes with MDM2 to enhance its function.

To determine whether there is a direct interaction between MDM2 and HBP1, we next performed a GST pulldown assay. GST-HBP1 pulled down MDM2 in vitro, whereas GST alone did not (Fig. 3c). Reciprocally, GST-MDM2 pulled down HBP1 in vitro, whereas GST alone did not (Fig. 3d). In summary, MDM2 directly interacts with HBP1 in vivo and in vitro. To further determine which domains were indispensable for the interaction of MDM2 with HBP1, we performed GST pulldown assays using a set of GST-tagged deletion mutants from HBP1 or MDM2. These data indicated that the HMG domain of HBP1 (designated HMG) did not interact with MDM2, whereas the other domains of HBP1 did. The stronger binding of HBP1 lacking HMG domain (designated ΔHMG) to MDM2 might be due to the deletion of the HMG domain induced conformational changes (Fig. 3c). Next we performed a reciprocal GST pulldown assay, the data indicated that the acidic region of MDM2 (designated AR) did not bind HBP1, whereas other mutants of MDM2 did (Fig. 3d). These results suggested that the interaction between MDM2 and HBP1 is dependent on diverse domains of the two proteins, but not on the HMG domain or the AR domain.

### MDM2 ubiquitinated HBP1 at K398

**Fig. 4:**
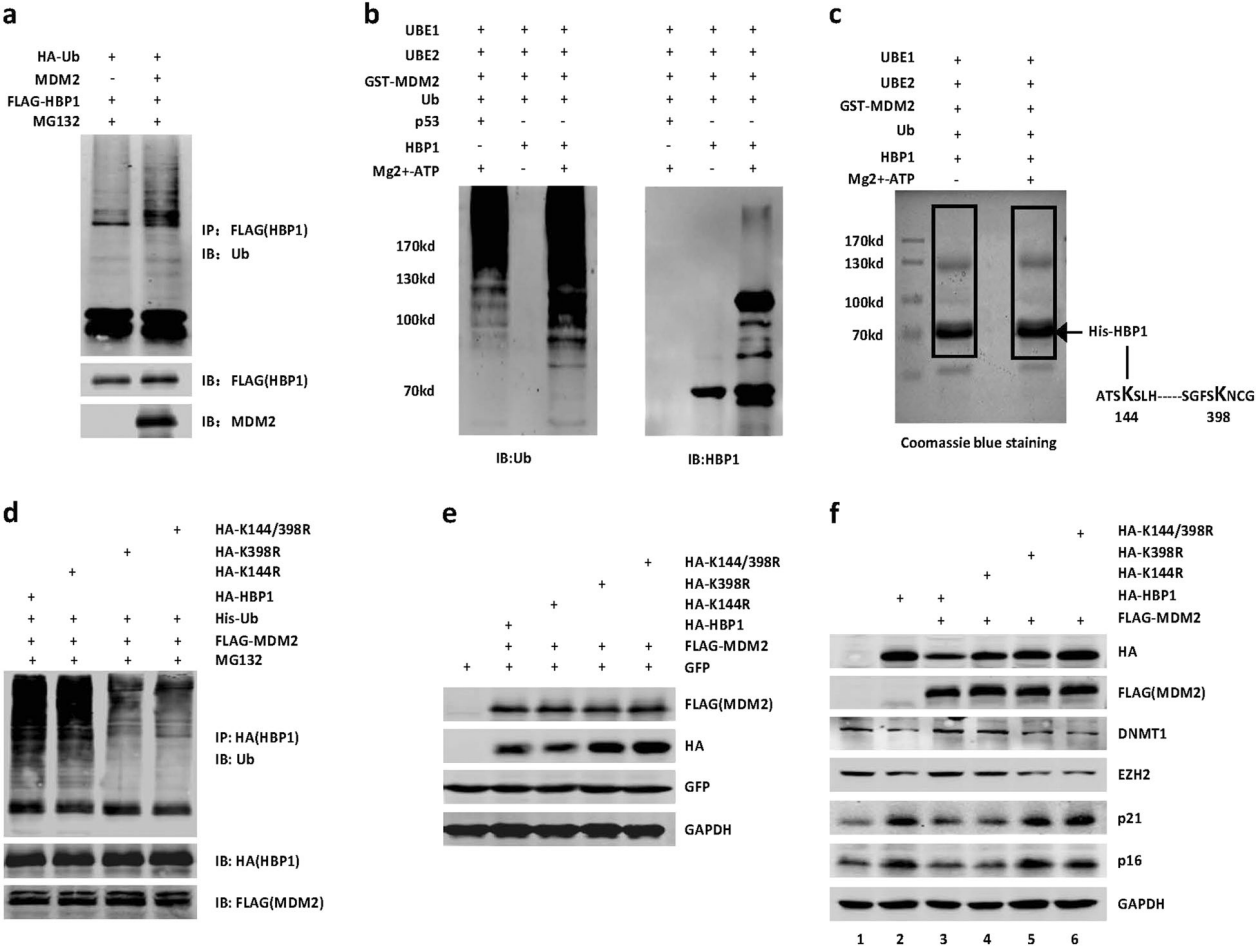
MDM2 ubiquitinates HBP1 at K398. **a** MDM2 ubiquitinates HBP1 in vivo. 293T cells were co-transfected FLAG-HBP1, HA-Ub with or without MDM2 for 18 h and then exposed to MG132 for another 6 h prior to lysis. HBP1 protein was then isolated by immunoprecipitation and analyzed by anti-Ub antibody. **b** MDM2 ubiquitinates HBP1 in vitro. Purified His-tagged HBP1 protein was incubated with or without ATP in the presence of UbE1, UbE2, GST-MDM2, and Ub. The reaction products were separated by SDS-PAGE and immunoblotted with the anti-Ub (left panel) and anti-HBP1 (right panel) antibody. His-p53 was used as a positive control. **c** Purified His-tagged HBP1 protein was incubated with or without ATP in the presence of UbE1, UbE2, GST-MDM2, and Ub. The reaction products were separated by SDS-PAGE and gels were stainned with Coomassie blue. The protein bands were retrieved and analyzed by mass spectrometry. **d** MDM2 ubiquitinates HBP1 at K398 in vivo. 293T cells were co-transfected HA-HBP1, HA-K144R, HA-K398R, or HA-K144/K398R with FLAG-MDM2 and His-Ubiquitin for 18 h and then exposed to MG132 for another 6 h prior to lysis. HBP1 protein was then isolated by immunoprecipitation and analyzed by anti-Ub antibody. **e** The decrease of HBP1 protein level induced by MDM2 depends on K398 ubiquitination. The protein levels of HBP1 and MDM2 were measured by western blotting in HeLa cells co-transfected HA-HBP1, HA-K144R, HA-K398R, or HA-K144/K398R with FLAG-MDM2 and GFP. Level of GFP was shown as equal transfection efficiency. GAPDH was used as a loading control. **f** MDM2 rescues the effect of HBP1 on expressions of its target genes. The protein levels of DNMT1, EZH2, p16, and p21 were measured by western blotting in H1299 cells co-transfected HA-HBP1, HA-K144R, HA-K398R, or HA-K144/K398R with or without FLAG-MDM2. GAPDH was used as a loading control

Since MDM2 is an E3 ubiquitin ligase, we tested whether HBP1 is ubiquitinated by MDM2. 293T cells were co-transfected with HBP1 and MDM2 or PRK5 (as a control), then exposed to MG132 for 6 h. Subsequently, HBP1 protein was isolated by immunoprecipitation, separated by SDS-PAGE and analyzed with an anti-Multi Ubiquitin (Ub) antibody. As shown in Fig. 4a, exogenous HBP1 was ubiquitinated in MDM2-expressing cells.

We also investigated HBP1 ubiquitination in vitro. The purified His-tagged HBP1 and E1 enzyme, E2 enzyme, GST-MDM2, ubiquitin were incubated with or without ATP. The reaction products were analyzed by western blotting using anti-Ub antibody, and the same blot was reprobed by anti-HBP1 antibody. These results further demonstrated that HBP1 was ubiquitinated by MDM2 in vitro (Fig. 4b). To identify the exact amino acids of ubiquitination, we next performed the in vitro ubiquitination assay and mass spectrometry. As shown in Fig. 4c, S1, K144 and K398 of HBP1 were ubiquitinated in the presence of MDM2. To confirm these results, we constructed three ubiquitination-deficient mutants: K144R, K398R, and K144/398R (Fig. 4d). Wild-type HBP1 was ubiquitinated by MDM2, consistent with previous data. One of the HBP1 single-residue mutants, K144R, was still ubiquitinated by MDM2, and to similar levels as wild-type HBP1, whereas for the other two mutants, K398R and K144/398R, ubiquitinated HBP1 levels were dramatically decreased, suggesting that MDM2 ubiquitinates HBP1 at K398.

Because MDM2 decreased HBP1 protein levels, we next explored whether HBP1 ubiquitination at K398 is required for MDM2-mediated decreased HBP1 protein levels. MDM2 decreased the protein levels of wild-type HBP1 and K144R but did not reduce the protein levels of K398R and K144/398R, suggesting that MDM2 decreased HBP1 protein levels by ubiquitinating HBP1 at K398 (Fig. 4e). We next investigated the effect of HBP1 ubiquitination on the regulation of its target genes. To this end, we co-transfected MDM2 expression plasmid with either wild-type HBP1 or the three ubiquitination-deficient mutants of HBP1 into H1299 cells. As shown in Fig. 4f, expressing wild-type HBP1 alone repressed DNMT1 and EZH2 protein levels and promoted p21 and p16 protein levels (lane 2), as previously reported [27–29], but the addition of MDM2 reduced HBP1 induction (lane 3). MDM2 addition also eliminated the induction of target genes by the ubiquitination mutant K144R (lane 4), while there was no effect on the other two ubiquitination mutants K398R and K144/398R (lanes 5, 6), which was consistent with data from expressing wild-type HBP1 alone.

These data indicated that HBP1 ubiquitination at K398 by MDM2 eliminated the transcriptional repression of its target genes, such as DNMT1 and EZH2, thereby decreasing p21 and p16 expression through enhanced DNA and histone methylation at promoter regions. DNMT1 and EZH2 are important methyltransferases that catalyze DNA and histone H3K27 trimethylation (H3K27me3), respectively, which are involved in genome instability. The CDK inhibitors p21 and p16 antagonize genome instability by regulating cell cycle progression. Thus, we hypothesized that MDM2 might induce genome instability by targeting HBP1 for proteasomal degradation in the absence of p53.

### MDM2-induced genomic instability by ubiquitinating HBP1

**Fig. 5:**
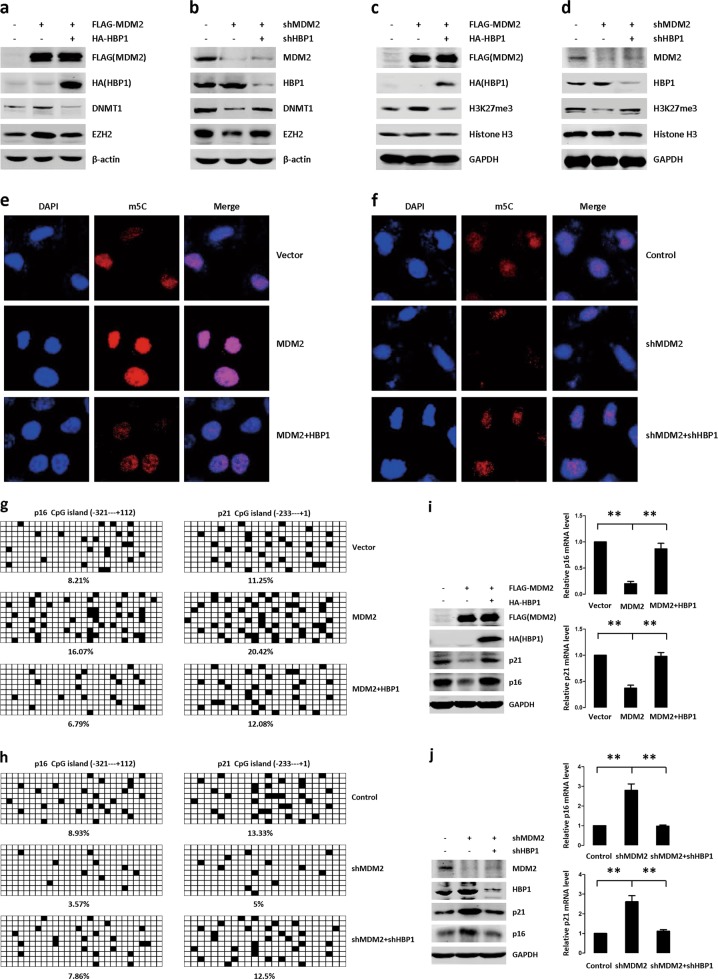

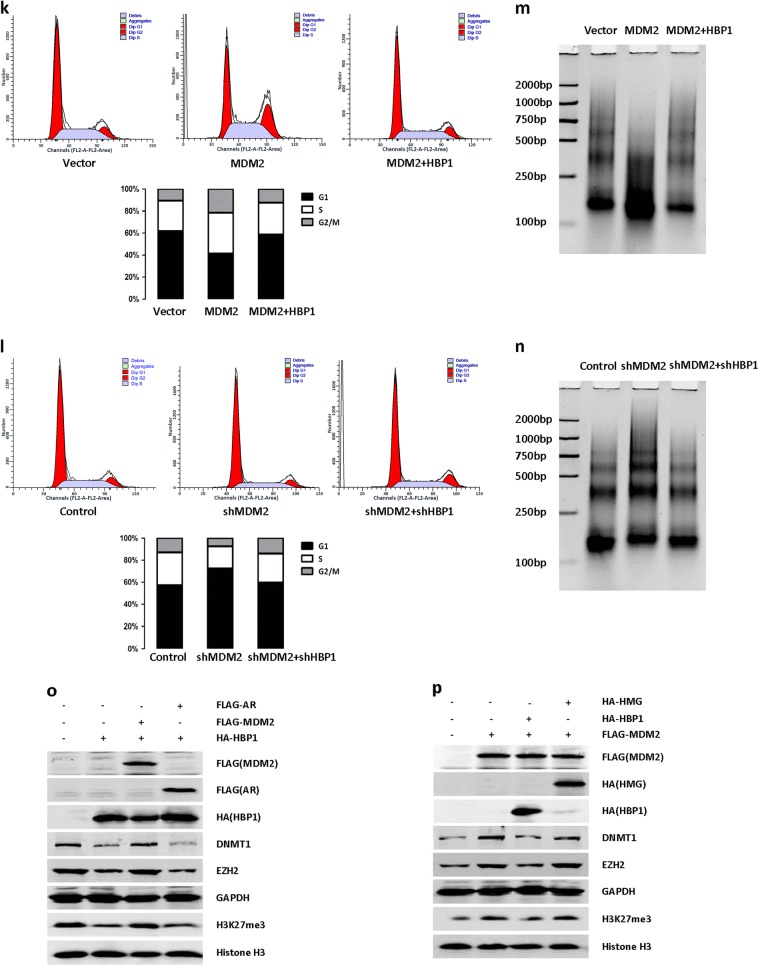

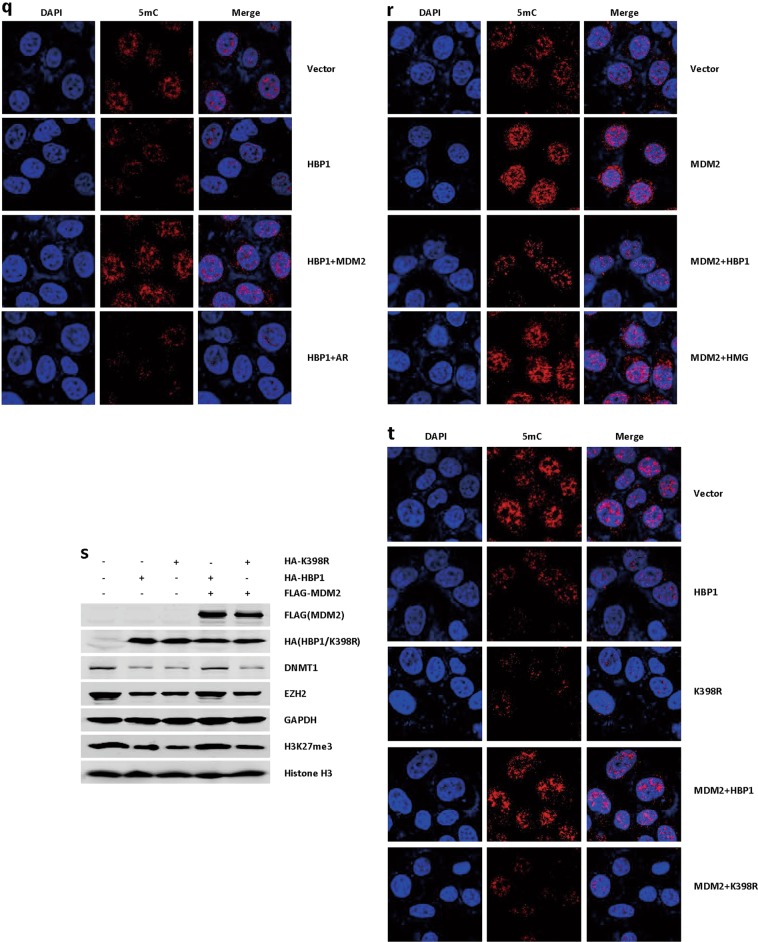
MDM2 induces genomic instability by ubiquitinating HBP1. **a** and **c** HBP1 overexpression rescues MDM2-inducing the upregulation of protein levels of DNMT1, EZH2 and H3K27me3. H1299 cells were co-transfected MDM2 with or without HBP1 expression plasmid. The protein levels of MDM2, HBP1, DNMT1, EZH2, H3K27me3, and histone H3 were measured by western blotting. Levels of β-actin or GAPDH were used as loading controls. **b** and **d** HBP1 knockdown rescues MDM2 knockdown-inducing the downregulation of protein levels of DNMT1, EZH2, and H3K27me3. H1299 cells were stably transfected with MDM2shRNA with or without HBP1shRNA. The protein levels of MDM2, HBP1, DNMT1, EZH2, H3K27me3, and histone H3 were measured by western blotting. Levels of β-actin or GAPDH were used as loading controls. **e** and **f** HBP1 overexpression rescues MDM2-inducing global DNA hypermethylation and HBP1 knockdown rescues MDM2 knockdown-inducing global DNA hypomethylation. Cells described in Fig. 5a, b were stained with antibodies specific for the 5mC epitope (red). **g** and **h** Bisulfite sequencing analysis was performed in the CpG islands in the promoters of the *p16* and *p21* genes in H1299 cells were co-transfected MDM2 with or without HBP1 **g**, or in H1299 cells were stably transfected MDM2shRNA with or without HBP1shRNA **h**. For each sample, 10 separate clones were chosen for sequencing. Symbols: □, unmethylated cytosine; ■, methylated cytosine. **i** HBP1 overexpression rescues MDM2-inducing the downregulation of mRNA and protein levels of p16 and p21. H1299 cells were co-transfected MDM2 with or without HBP1. The protein levels of p16 and p21 were measured by western blotting. Level of GAPDH was used as a loading control (left panel). The mRNA levels of p16 and p21 were measured by Real-time PCR (right panel). The mean ± S.D. for three independent experiments are shown. **p < 0.01. **j** HBP1 knockdown rescues MDM2 knockdown-inducing the upregulation of mRNA and protein levels of p16 and p21. H1299 cells were stably transfected MDM2shRNA with or without HBP1shRNA plasmid. The protein levels of p16 and p21 were measured by western blotting. Level of GAPDH was used as a loading control (left panel). The mRNA levels of p16 and p21 were measured by real-time PCR (right panel). The mean ± S.D. for three independent experiments are shown. **p < 0.01. **k** and **l** HBP1 overexpression rescues MDM2-inducing the promotion of cell cycle **k** and HBP1 knockdown rescues MDM2 knockdown-inducing the arrest of cell cycle **l**. Cells described in Fig. 5i, j were subjected to FCS analyzed. Data are representative from three-independent experiments. **m** and **n** HBP1 overexpression rescues MDM2-inducing the higher sensitivity to micrococcal nuclease (MNase) digestion(m) and HBP1 knockdown rescues MDM2 knockdown-inducing the lower sensitivity to micrococcal nuclease (MNase) digestion(n). Cells described in Fig. 5i, j were digested with MNase for 5 min at 25 °C, and the genomic DNA was subsequently extracted and separated by a 6% PAGE gel. **o** H1299 cells were co-transfected HBP1 with or without MDM2 or AR expression plasmid. The protein levels of MDM2, HBP1, DNMT1, EZH2, H3K27me3, and histone H3 were measured by western blotting. Level of GAPDH was used as loading control. **p** H1299 cells were co-transfected MDM2 with or without HBP1 or HMG expression plasmid. The protein levels of MDM2, HBP1, DNMT1, EZH2, H3K27me3, and histone H3 were measured by western blotting. Level of GAPDH was used as loading control. **q** and **r** Cells described in Fig. 5o, p were stained with antibodies specific for the 5mC epitope (red). **s** H1299 cells were co-transfected HBP1 or K398R with or without MDM2 expression plasmid. The protein levels of MDM2, HBP1, DNMT1, EZH2, H3K27me3, and histone H3 were measured by western blotting. Level of GAPDH was used as loading control. **t** Cells described in Fig. 5s were stained with antibodies specific for the 5mC epitope (red)

We used a lentiviral expression vector to overexpress MDM2 and/or HBP1 proteins in H1299 cells. While excess MDM2 increased DNMT1, EZH2, and H3K27me3 levels, co-expressing HBP1 rescued the MDM2-mediated increases in DNMT1, EZH2, and H3K27me3 (Fig. 5a, c). Furthermore, shRNA knockdown of MDM2 increased HBP1 expression, thereby decreasing DNMT1, EZH2, and H3K27me3 levels, but there was no effect when HBP1 was also knocked down (Fig. 5b, d). These results indicated that MDM2 and HBP1 act together to regulate DNMT1, EZH2, and H3K27me3, and that the actions of MDM2 require HBP1.

DNMT1 has been reported to play a key role in regulating global DNA methylation and the methylation of the p21 and p16 promoters [29]. Thus, we next asked if the MDM2/HBP1/DNMT1 axis regulates global DNA methylation and/or specific sites within the p21 and p16 promoters, thereby interfering with p21 and p16 expression and genome instability. To investigate the relationship between overall methylated DNA levels and MDM2 expression levels, we measured 5-methylcytosine (5mC). (Fig. 5e, f). We expressed MDM2, MDM2+HBP1, MDM2shRNA, or MDM2shRNA+HBP1shRNA in H1299 cells individually through lentiviral infection. After selection, we co-stained for 5mC. MDM2-infected cells showed strong staining for 5mC, co-expressing HBP1 rescued the MDM2-mediated strong 5mC staining (Fig. 5e), indicating that MDM2-induced DNA hypermethylation, while HBP1 expression rescued hypermethylation. Furthermore, MDM2shRNA-infected cells showed decreased 5mC staining, while co-expressing HBP1shRNA rescued this decreased 5mC staining (Fig. 5f). Together, these data indicated that MDM2 induces global DNA hypermethylation by decreasing HBP1 expression and thus increasing DNMT1 expression.

We next investigated the reciprocal relationship between MDM2 and HBP1 with regard to the methylation state of *p21* and *p16*. Similar results were obtained by bisulfate sequencing analysis of the *p21* and *p16* promoters in H1299 cells infected the same plasmids as above. Upon MDM2 overexpression, the methylation levels of *p21* and *p16* promoter increased to 16.07% and 20.42% of CGs, respectively, suggesting hypermethylation. Again, in cells doubly expressing MDM2 and HBP1, the methylation levels of *p21* and *p16* promoter were restored to 6.79% and 12.08% of CGs, respectively, which were near control levels (8.21% and 11.25%, respectively) (Fig. 5g). Furthermore, MDM2 knockdown by shRNA decreased *p21* and *p16* promoter methylation levels, whereas shRNA knockdown of HBP1 rescued MDM2 knockdown-induced hypomethylation (Fig. 5h).

We also tested whether the MDM2/HBP1/DNMT1 axis regulates p16 and p21 expression. We had previously reported that hypomethylation of the *p16* and *p21* promoter, which was attributed to HBP1 repressing DNMT1, increased p16 and p21 protein levels [29]. Thus, we next tested effects of MDM2 repression on HBP1. By real-time PCR and western blotting, MDM2 overexpression decreased p16 and p21 mRNA and protein levels, while co-expressing HBP1 rescued the MDM2-mediated decreases in p16 and p21 expression (Fig. 5i). Furthermore, shRNA knockdown of MDM2 increased p16 and p21 mRNA and protein levels, but had no effect if HBP1 was also knocked down (Fig. 5j). Together, these results indicated that the MDM2/HBP1/DNMT1 axis regulates global DNA methylation and the specific promoter methylation of *p21* and *p16*, thereby interfering with p21 and p16 expression.

As p16 and p21 inhibit cell cycle progression, the decreases in p16 and p21 induced by MDM2 caused an increase in the S and G2/M populations, whereas HBP1 overexpression rescued these effects (Fig. 5k). MDM2 knockdown with or without HBP1 knockdown by shRNA caused the opposite results (Fig. 5l). Additionally, MDM2 overexpression decreased genomic stability (Fig. 5m), as assessed by genomic stability assay, whereas MDM2 knockdown by shRNA increased genomic stability (Fig. 5n). HBP1 overexpression attenuated MDM2-inducing genomic instability (Fig. 5m), whereas HBP1 knockdown by shRNA attenuated MDM2 knockdown-induced genomic stability (Fig. 5n). These results suggested that MDM2 induces cell cycle progression and genomic instability through its ability to ubiquitinate HBP1.

We also tested whether the interaction of HBP1 and MDM2 is required for regulation of genome stability. We overexpressed HBP1, HBP1+MDM2, HBP1+AR (AR, a MDM2 mutant that fails to bind to HBP1), MDM2, MDM2+HBP1, MDM2+HMG (HMG, a HBP1 mutant that fails to bind to MDM2) in H1299 cells individually. As shown in Fig. 5o, q, co-expressing MDM2 reversed the HBP1-induced decreases in DNMT1, EZH2, H3K27me3 (Fig. 5o), and 5mC staining (Fig. 5q), while AR mutant had no effect. Accordingly, HBP1 reversed the MDM2-induced increases in DNMT1, EZH2, H3K27me3 (Fig. 5p), and 5mC staining (Fig. 5r), while HMG mutant had no effect. Furthermore, HBP1 induced a significant decrease in DNMT1, EZH2, H3K27me3 (Fig. 5s) and 5mC staining (Fig. 5t), K398R mutant could also induce the same results. MDM2 reversed HBP1-induced decreases in DNMT1, EZH2, H3K27me3 (Fig. 5s) and 5mC staining (Fig. 5t), but had no effect on K398R induction. These results indicated that the interaction of HBP1 and MDM2 is indispensable for regulation of genome stability. HBP1 ubiquitination by MDM2 reduced its promotion in genome stability.

### The MDM2/HBP1 axis facilitated MDM2-induced tumorigenesis

**Fig. 6:**
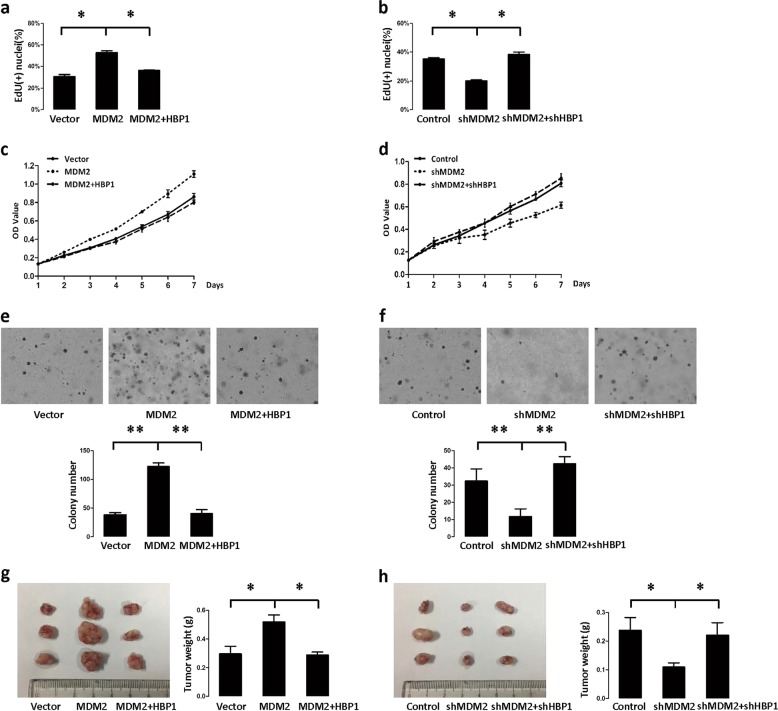
The MDM2/HBP1 axis facilitates MDM2-induced tumorigenesis. **a** and **c** HBP1 overexpression rescues MDM2-inducing increase in cell growth. EdU incorporation **a** and MTT **c** assays were conducted with H1299 cells stably transfected with control vector, MDM2, or MDM2+HBP1. The mean ± S.D. for three-independent experiments are shown. *p < 0.05. **b** and **d** HBP1 knockdown rescues MDM2 knockdown-inducing decrease in cell growth. EdU incorporation **b** and MTT **d** assays were conducted with H1299 cells stably transfected with control vector, MDM2shRNA, or MDM2shRNA+HBP1shRNA. The mean ± S.D. for three-independent experiments are shown. *p < 0.05. **e** HBP1 overexpression rescues MDM2-inducing increase of cell growth in soft agar, and **f** HBP1 knockdown rescues MDM2 knockdown-inducing decrease of cell growth in soft agar. Soft agar colony formation assay of the cells described in Fig. 6a, b. Cells were cultured in soft agar for 2 weeks (top panel). The colony numbers in three different microscope fields were counted and are shown as mean ± S.D. (bottom panel) **p < 0.01. **g** HBP1 overexpression rescues MDM2-inducing increase in tumorigenesis and **h** HBP1 knockdown rescues MDM2 knockdown-inducing decrease in tumorigenesis. Cells described in Fig. 6a, b were subcutaneously injected into nude mice. Four weeks after injection, the tumors were weighed, and size was measured. Data are shown as mean ± S.D. (*n* = 3). * *p* < 0.05

We overexpress MDM2 and/or HBP1 through lentiviral infection in H1299 cells. Consistent with previous studies [37], MDM2 increased proliferation, as shown by the EdU incorporation assay and growth curves (Fig. 6a, c). MDM2 also enhanced tumorigenesis, as demonstrated by increased anchorage-independent growth in soft agar (Fig. 6e) and xenograft tumorigenesis in nude mice (Fig. 6g). Both MDM2-induced S-phase promotion and increased growth rate were reversed by expressing HBP1 (Fig. 6a, c). Consequently, HBP1 also rescued MDM2-induced tumorigenesis (Fig. 6e, g). Next, MDM2 knockdown cells showed decreased EdU incorporation (Fig. 6b) and growth rate (Fig. 6d) and inhibited tumorigenesis (Fig. 6f, h). HBP1 knockdown by shRNA rescued the effects of MDM2 knockdown (Fig. 6b, d, f, h). Thus, the precise balance of MDM2 and HBP1 is critical for MDM2-induced tumorigenesis.

To determine whether HBP1 overexpression alters methylation and tumorigenesis in MDM2 high expression cell lines, MCF-7 and MDA-MB-231 cells were transfected with HBP1 expression vector. HBP1 decreased DNMT1, EZH2, and H3K27me3 levels, and 5mC staining (Fig. S2a, b). These results indicated that HBP1 induces DNA and histone hypomethylation in MDM2 high expression cell lines. Then we overexpressed HBP1 through lentiviral infection in MCF-7 and MDA-MB-231 cells. HBP1 decreased cell proliferation, as shown by the EdU incorporation assay and growth curves (Fig. S2c, d). HBP1 also inhibited tumorigenesis, as demonstrated by decreased anchorage-independent growth in soft agar (Fig. S2e) and xenograft tumorigenesis in nude mice (Fig. S2f). These results suggested that HBP1 inhibits cell proliferation and tumorigenesis in MDM2 high expression cell lines.

### Blocking MDM2-mediated HBP1 repression facilitated DNA repair after ionizing radiation-induced DNA damage

Christine et al. reported that MDM2 binds to Nbs1, a component of the M-R-N complex in primary cells and in p53-inactivated cells [38]. The M-R-N complex maintains DNA integrity through DNA double-strand break repair, meiotic recombination and telomere maintenance. Nbs1 is capable of aggregating of the M-R-N complex at DNA damage sites. MDM2 appears to regulate DNA damage repair by inhibiting the ability of the M-R-N complex, but the exact mechanisms are not understood. We hypothesized that MDM2-mediated HBP1 repression might interfere with DNA break repair and contribute to genome instability and tumorigenesis in a p53-independent manner.

**Fig. 7:**
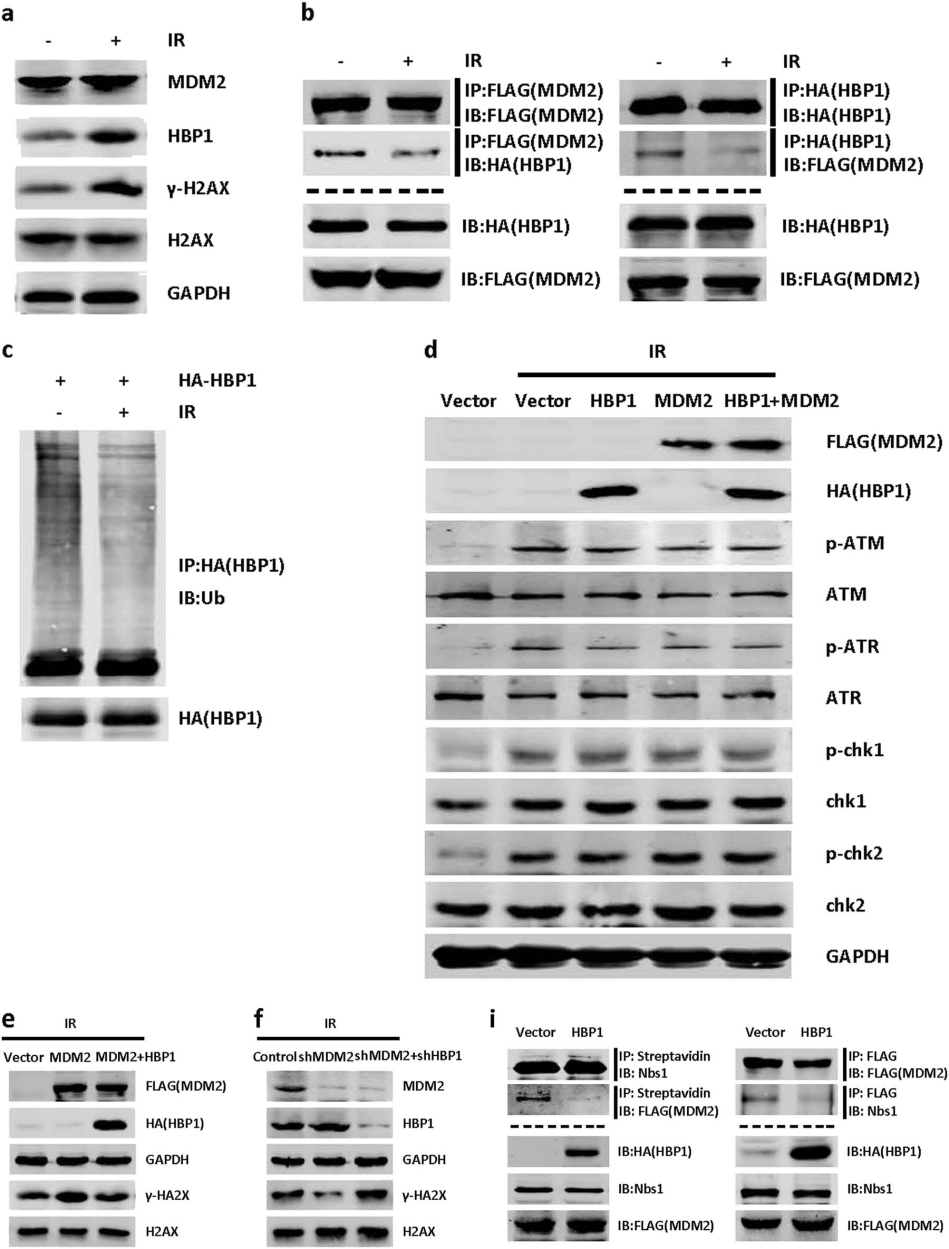

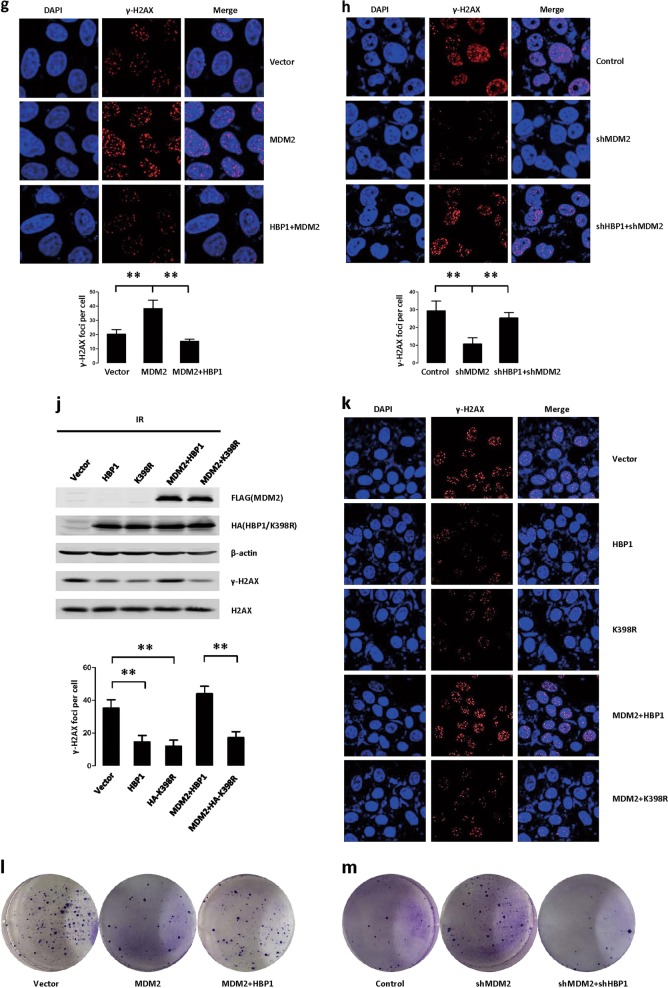
Blocking MDM2-mediated HBP1 repression facilitates DNA repair after ionizing radiation (IR)-induced DNA damage. **a** The inverse correlation between MDM2 and HBP1 protein levels in H1299 cells treated with ionizing radiation (10 Gy) and recovered for 3 h. The protein levels of MDM2, HBP1, γ-H2AX, H2AX were measured with western blotting. GAPDH was used as a loading control. **b** Ionizing radiation inhibits the interaction between MDM2 and HBP1.H1299 cells were co-transfected HA-HBP1 and FLAG-MDM2 with or without ionizing radiation (10 Gy) and recovered for 3 h, IP assay was carried out by using anti-FLAG/HA antibody and followed by western blotting with anti-HA/FLAG antibody. The same samples were immunoblotted against FLAG/HA to determine immunoprecipitation efficiency. **c** Ionizing radiation inhibits the ubiquitination of HBP1. H1299 cells were co-transfected HA-HBP1 and FLAG-MDM2 with or without ionizing radiation (10 Gy) and recovered for 3 h. HBP1 protein was then isolated by immunoprecipitation and analyzed by anti-Ub antibody. **d** H1299 cells were transfected with HBP1, MDM2 or HBP1+MDM2 expression plasmid were treated with ionizing radiation (10 Gy) and recovered for 30 min, the protein levels of p-ATM, p-ATR, p-chk1, and p-chk2 were measured with western blotting. GAPDH was used as a loading control. **e** HBP1 overexpression rescues MDM2-inducing increase in γ-H2AX following ionizing radiation, and **f** HBP1 knockdown rescues MDM2 knockdown-inducing decrease in γ-H2AX following ionizing radiation. H1299 cells stably transfected with control vector, MDM2, or MDM2+HBP1 **e**, or H1299 cells stably transfected with control vector, MDM2shRNA, or MDM2shRNA+HBP1shRNA **f**, were treated with ionizing radiation (3 Gy) and recovered for 24 h, the protein levels of γ-H2AX, H2AX were measured with western blotting. GAPDH was used as a loading control. **g** HBP1 overexpression rescues MDM2-inducing increase in number of γ-H2AX foci following ionizing radiation, and **h** HBP1 knockdown rescues MDM2 knockdown-inducing decrease in number of γ-H2AX foci following ionizing radiation. Cells described in Fig. 7e, f were stained with antibodies specific for the γ-H2AX epitope (red). The number of γ-H2AX foci per each cell was shown from 40 cells calculated. **p < 0.01. **i** HBP1 inhibits the interaction between MDM2 and Nbs1. H1299 cells were co-transfected SFB-Nbs1 and FLAG-MDM2 with or without HA-HBP1. The IP assay was carried out by using Streptavidin Sepharose and followed by western blotting with anti-HA antibody (left panel). H1299 cells were co-transfected FLAG-MDM2 with or without HA-HBP1. IP assay was carried out by using anti-FLAG antibody and followed by western blotting with anti-Nbs1 antibody (right panel). **j** and **k** HBP1 promotes DNA damage repair following ionizing radiation. H1299 cells were co-transfected HBP1 or K398R with or without MDM2 expression plasmid were treated with ionizing radiation (3 Gy) and recovered for 24 h, Western boltting **j** and γ-H2AX foci staining **k** assays were performed. GAPDH was used as a loading control. The number of γ-H2AX foci per each cell is shown from 40 cells calculated. **p < 0.01. **l** HBP1 overexpression rescues MDM2-inducing decrease in clonogenic cell survival following ionizing radiation, and **m** HBP1 knockdown rescues MDM2 knockdown-inducing increase in clonogenic cell survival following ionizing radiation. Cells described in Fig. 7e, f were seeded into a 6-well plate. Cells were fixed and stained with crystal violet after 14 days

To investigate the effects of MDM2 and HBP1 on DNA damage repair, we detected the levels of γ-H2AX. After DNA damage, histone H2AX is rapidly phosphorylated at Ser139 (becoming γ-H2AX), and as the DNA is gradually repaired, γ-H2AX levels are reduced [36]. Therefore, levels of γ-H2AX reflect the degrees of response to DNA damage or DNA damage repair. Immediately following ionizing irradiation, H1299 cells had more γ-H2AX than the control cells without ionizing radiation, indicating that ionizing radiation-induced DNA damage. Interestingly, HBP1 protein also increased following ionizing radiation, while MDM2 protein was unchanged with or without ionizing radiation (Fig. 7a). Next, we sought to determine whether ionizing radiation inhibits MDM2-mediated HBP1 ubiquitination. As shown in Fig. 7b, ionizing radiation disrupted the interaction between MDM2 and HBP1. Accordingly, HBP1 ubiquitination was decreased in H1299 cells treated with ionizing radiation (Fig. 7c). Furthermore, we tested whether the MDM2 or HBP1 could interfere pATR-pCHK1/pATM-pCHK2 signal pathway, which is upstream of MDM2 that is activated following ionizing radiation. As shown in Fig. 7d, pATR-pCHK1/pATM-pCHK2 signal pathway was activated following ionizing radiation, but overexpression of HBP1, MDM2 or co-expression of HBP1 and MDM2 individually did not interfere the activation of pATR-pCHK1/pATM-pCHK2 signal pathway following ionizing radiation. We then co-transfected MDM2, or both MDM2 and HBP1 into H1299 cells, and knocked down MDM2, or both MDM2 and HBP1 through lentiviral infection. We collected cells 24 h after treatment with ionizing radiation, and detected the levels of γ-H2AX by western blotting. As shown in Fig. 7e, MDM2 expression increased γ-H2AX protein levels, while HBP1 reversed the MDM2-induced increase in γ-H2AX. Furthermore, MDM2 knockdown by shRNA decreased γ-H2AX levels but if HBP1 was also knocked down, γ-H2AX levels were no longer affected (Fig. 7f). The data indicated that overexpressing MDM2 delays DNA break repair by blocking HBP1 expression.

The quantification of γ-H2AX foci following ionizing radiation revealed that MDM2-overexpressing H1299 cells had significantly greater numbers of γ-H2AX foci than vector controls (Fig. 7g). In the presence of HBP1, the numbers of γ-H2AX foci were restored to control levels, eliminating the effect of MDM2. Accordingly, shRNA knockdown of MDM2 decreased the number of γ-H2AX foci but if HBP1 was also knocked down, the number ofγ-H2AX foci was no longer affected (Fig. 7h). These results also confirmed the conclusion that the MDM2 repression of HBP1 through ubiquitination affects DNA break repair following ionizing radiation.

Additionally, it has been found that MDM2 can suppress DNA break repair by binding Nbs1 and destroying the M-R-N complex [25]. Thus, we next investigated whether HBP1 interferes with the binding of MDM2 and Nbs1, thus contributing to DNA break repair. As shown in Fig. 7i, overexpressing HBP1 inhibited MDM2 binding to Nbs1. Furthermore, HBP1 induced a significant decrease in γ-H2AX protein level (Fig. 7j) and the number of γ-H2AX foci following ionizing radiation (Fig. 7k). K398R mutant also induced the same results after ionizing radiation. MDM2 reversed HBP1-induced decrease in γ-H2AX protein level and the number of γ-H2AX foci, but had no effect on K398R induction (Fig. 7j, k). These data suggested that HBP1 promotes DNA break repair and maintains genome stability by inhibiting MDM2 binding to Nbs1, thus promoting the effects of the M-R-N complex on DNA damage sites. HBP1 ubiquitination by MDM2 reduced its promotion in DNA break repair.

Finally, we used lentivirus to overexpress MDM2, MDM2+HBP1, MDM2shRNA, and MDM2shRNA+HBP1shRNA in H1299 cells individually. Then the transfected cells were treated with ionizing radiation. As shown in Fig. 7l, MDM2 overexpression-induced cell death following ionizing radiation, as demonstrated by decreased numbers of cell colonies, whereas HBP1 overexpression abolished the cell death induced by MDM2. Furthermore, MDM2 knockdown by shRNA induced cell growth following ionizing radiation, whereas HBP1 knockdown abolished the cell growth induced by MDM2 knockdown (Fig. 7m). Together, these results suggested that MDM2 overexpression-mediated repression of HBP1 delays DNA break repair and causes cell death in a p53-independent manner. Blocking the repression of HBP1 by MDM2 facilitates DNA repair after ionizing radiation.

## Discussion

**Fig. 8:**
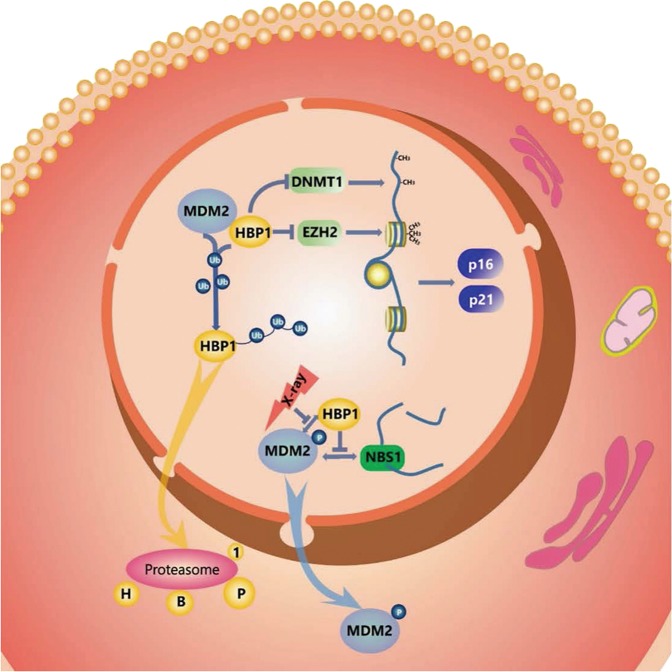
Model for the proposed role of MDM2/HBP1 axis in regulating genome instability and DNA damage repair. MDM2 inhibits HBP1 protein expression by binding and ubiquitinating at the position of K398, thus abrogating HBP1-mediated transcriptional inhibition of DNMT1 and EZH2. This leads to global DNA and histone hypermethylation, which selectively turns off *p16* and *p21* transcriptions, resulting in an increase in cell cycle progression and further facilitating genome instability and tumorigenesis. MDM2-mediated repression of HBP1 also delays DNA damage repair and causes genome instability following ionizing radiation. Overall, MDM2 promotes genome instability by ubiquitinating the transcription factor HBP1

The data in this paper support two conclusions that are represented in the model (Fig. 8). First, the data demonstrate that transcription factor HBP1 is a target of MDM2. MDM2 interacts and regulates the stability of HBP1 through ubiquitination. With mass spectrometry and ubiquitination-deficient mutants, the experiments identified that HBP1 ubiquitination at K398 is required for MDM2-mediated decreased HBP1 protein levels. Given that HBP1 is a tumor suppressor, MDM2 might affect tumorigenesis and response to oncogenic growth factor signaling by downregulation of HBP1. Second, the data support a role for the MDM2/HBP1 axis in regulating genome instability and tumorigenesis. MDM2 increased HBP1 proteasomal degradation by ubiquitination, thus decreasing HBP1 protein levels and attenuating the transcriptional inhibition of HBP1 target genes, such as *DNMT1* and *EZH2*, which results in DNA and histone hypermethylation and genome instability. The repression of HBP1 by MDM2 ultimately induces cell growth and tumorigenesis (Figs. 1–6). Furthermore, MDM2 regulation of HBP1 participates in DNA damage repair following ionizing radiation. Using ubiquitination and γ-H2AX foci assays, we showed that the ability of MDM2 to ubiquitinate HBP1 decreased following ionizing radiation. The increased HBP1 levels inhibited MDM2 binding to Nbs1, thus facilitating the M-R-N complex to promote double-strand break repair and maintain genome stability. MDM2 overexpression delayed double-strand break repair and induced genome instability and cell death by decreasing HBP1 levels (Fig. 7). Together, these data indicate that MDM2 interferes with genome stability independent of p53 and further confirms HBP1 as a mediator of genome stability. The balance in the MDM2-HBP1 partnership can affect genome stability and/or trigger cell death or tumorigenesis.

Maintaining genome integrity is essential for preventing transformation, and several reports have provided evidence that MDM2 levels are positively correlated with genome instability and tumorigenesis [8, 39–41]. These studies have illustrated that decreasing MDM2 levels reduces chromosomal instability, while increasing MDM2 expression results in an increase in genomic instability. However, the p53-independent roles of MDM2 in genome stability remained elusive.

This study adds HBP1 as a functionally relevant player in maintaining genome stability. HBP1 was originally identified as a tumor inhibitor and a p38 MAPK-inducible protein [42]. We previously demonstrated that HBP1 causes global DNA hypomethylation and decreases H3K27me3 through the transcriptional repression of *DNMT1* and *EZH2*, thus maintaining genome stability and inducing premature senescence in response to transgenic *Ras* or *Pim-1* in human diploid fibroblasts [28, 43]. This indicated that high HBP1 activity creates a barrier to tumorigenesis. In this study, we showed that MDM2 targets and inhibits HBP1 via proteasomal degradation. This non-canonical MDM2 function could contribute to genome instability and further promote tumorigenesis.

DNA damage can be induced by various external stimuli, such as ionizing radiation, UV exposure, oxidative stress, and chemotherapeutic drugs [44]. To prevent genome instability, a set of mechanisms that can sustain genome stability were established by cells. Upon DNA damage, MDM2 binds to Nbs1, which constitutes a DNA damage response that aids the M-R-N complex, thereby inhibiting the DNA damage response by preventing the M-R-N complex from associating with damaged DNA [25]. MDM2 is inhibited by phosphorylation following ionizing radiation, which could promote its translocation from the nucleus to the cytoplasm, thereby blocking its function on the M-R-N complex [45, 46]. After DNA damage, the MDM2-HBP1 interaction is inhibited, possibly due to MDM2 translocation to the cytoplasm. Importantly, HBP1 could inhibit the interaction between MDM2 and Nbs1, relieving the repression of the M-R-N complex. In the course of DNA damage responses, both DNA replication and cell cycle progression are arrested by the elevated p53 expression. This causes the widespread alteration of downstream genes, and some types of DNA damage can only be repaired when DNA replication is suspended [47]. HBP1 blocks cell cycle progression by increasing p16 and p21 expression independent of p53, thus providing the necessary conditions for the DNA damage response pathway to be active. Additionally, we have discovered that MDM2 and HBP1 impact genome stability. HBP1 makes chromatin tighter by inhibiting the expression of DNMT1 and EZH2, and reducing DNA damage during external stimulation.

Our studies place MDM2 and HBP1 at an important junction regarding genome instability and suggest that these two proteins may be part of the “genome instability-executing machinery” that may be interrupted by ionizing radiation and other signals. If this MDM2/HBP1 axis were augmented, then a cascade of events leading to tumorigenesis could be initiated. MDM2 is a transcriptional target of MYC, which is frequently overexpressed in human cancers and as a central oncogene/driver in many cancers [48]. Also, MYC maintains a neoplastic state through the regulation of microRNA cluster miR-17-92, which controls HBP1 levels [49]. So we speculate that MDM2/HBP1 axis might participate in MYC regulatory pathway and contribute to MYC-induced tumorigenesis through suppression of downstream targets of p21 and p16.

As an oncoprotein, MDM2 could contribute DNA damage repair dysfunction and promote cell death by repressing HBP1, which makes it a potential target for cancer therapy. The mechanisms of the MDM2/HBP1 axis-mediated DNA methylation and histone methylation are required to promote genome instability and tumorigenesis in the absence of p53. We propose that the HBP1-dependent functions of MDM2 should be considered in the design of anti-cancer drugs. So far, the majority of MDM2 inhibitors target their binding to p53 in clinical trials but do not affect the function of MDM2 described here [50, 51]. However, when MDM2 inhibitors targeting the E3 ligase activity of MDM2, its broader carcinogenic activity might become pharmaceutically acceptable. Recent studies have demonstrated that combination treatment with MDM2 inhibitor and Bcl-2 inhibitor results in synergistic anti-tumor activity compared with the respective single-agent treatments in acute myeloid leukemia and colon cancer cells [52, 53]. These findings provide functional and molecular insight on the superior anti-tumor activity of combined MDM2 and Bcl-2 inhibitors in cancer cells and offer further options for therapy.

The identification of MDM2 overexpression leading to an inhibition of DNA break repair and causing cell death by repressing HBP1 also opens up new therapeutic avenues for cancer therapy. We have previously reported that HBP1 is increased and that elevated HBP1 promotes premature senescence or apoptosis to avoid undergoing tumorigenesis in H_2_O_2_-stimulated cells [43]. In this work, we underscore the importance of the MDM2/HBP1 axis in regulating the DNA damage repair in response to DNA damage. From the cancer standpoint, we predict that combining cell cycle arrest with DNA damage repair via the MDM2/HBP1 axis might have therapeutic potential for human cancer. This is especially important for malignant tumors caused by lost function of p53. Future research should focus on further characterizing the effects of elevated MDM2 and HBP1 levels on DNA damage for therapeutic purposes.

## Methods and materials

### Cell culture, transfection, and lentivirus infection

293T, HeLa, and H1299 cell lines were cultured in DMEM containing 10% fetal bovine serum (FBS) at constant temperature of 37 °C. Cell transfection were utilizing TurboFect transfection reagent (Thermo Scientific) referring to the manufacturer’s instruction. The transfection efficiency was confirmed after 48 h post transfection. The lentivirus plasmid pLL3.7-shHBP1 could express shRNA targeting HBP1 mRNA (5′-ACTGTGAGTGCCACTTCTC-3′) and the lentivirus plasmid pLL3.7-shMDM2 could expresse shRNA targeting MDM2 mRNA (5′-GCTTCGGAACAAGAGACTC-3′).

### Immunohistochemistry

Tissue microarray slides were dewaxed, antigen retrieved and fixed in hydrogen peroxide/methanol solution. Then tissue slides were incubated with primary antibodies, followed by biotin labeled secondary antibodies, and colored by streptavidin ALP system. Stained areas were quantitated with Image J software.

### Western blotting and antibodies

Cells were collected and lysed in RIPA buffer (Thermo Scientific) mixing with protease inhibitor cocktail (Sigma), the protein concentrations examination were utilizing the BCA protein assay kit (Pierce). We detected protein expression utilizing 20–60 μg of samples, samples were separated by SDS-PAGE and transferred to nitrocellulose membranes (Pall). The primary antibodies for western blotting were as follows: anti-HBP1 (11746-1-AP, Proteintech), anti-MDM2 (sc-965, Santa Cruz Biotechnology), anti-p21(K0081-3, MBL), anti-p16(10883-1-AP, Proteintech), anti-DNMT1(sc-10222, Santa Cruz Biotechnology), anti-EZH2(21800-1-AP, Proteintech), anti-FLAG (F1804, Sigma-Aldrich), anti-HA(MMS101P, Covance), anti-Muti-Ub(D058-3, MBL), anti-γ-H2AX(ab26350, Abcam), and anti-GAPDH(KM9002, Sungene). The fluorescence labeled secondary antibodies we used were as follows: anti-mouse IgG antibody DyLight 800 (610-145-121, Rockland) and anti-rabbit IgG DyLight 800 (611-145-002, Rockland). The infrared fluorescence graphic results were acquired utilizing Odyssey infrared imaging system (LI-COR Bioscience, Lincoln, NE).

### Real-time PCR

Total RNA extraction was utilizing the RNAsimple Total RNA kit (Tiangen). Reverse transcription was performed utilizing ReverAid FirstStrand cDNA Synthesis kit (Thermo Scientific). The mRNA expression level was detected by real-time PCR utilizing Maxima SYBR Green qPCR Master Mix (Thermo Scientific). The primers’ sequences were shown as follows: human HBP1, 5′-TGAAGGCTGTGATAATGAGGAAGAT-3′ and 5′-CATAGAAAGGGTGGTCCAGCTTA-3′; human MDM2, 5′-GCAGTGAATCTACAGGGACGC-3′ and 5′-ATCCTGATCCAACCAATCACC-3′; and human GAPDH, 5′-CCATGGAGAAGGCTGGGG-3′ and 5′-CAAAGTTGTCATGGATGACC-3′. The mRNA expression was normalized by GAPDH.

### Immunoprecipitation

Cells were collected and lysed in IP lysis buffer (25 mM Tris-HCl (pH 7.4), 150 mM NaCl, 1% Nonidet P-40, 1 mM EDTA, and 5% glycerol) mixing with protease inhibitor cocktail (Sigma). The lysates were incubated with primary antibodies or control IgG, in addition with protein A-Sepharose (GE Healthcare) or Streptavidin Sepharose (GE Healthcare) overnight at 4 °C in rotation incubator. Samples were washed with IP lysis buffer for three times. We then examined relevant protein binding utilizing western blotting.

### GST pulldown assay

The purified GST-tagged proteins or GST alone and glutathione-Sepharose beads (GE Healthcare) were incubated together overnight at constant temperature of 4 °C. Then the His-tagged proteins were added and also incubated for 4 h at 4 °C. To avoid unspecified binding, we used binding buffer wash the beads for three times and used GST elution buffer to elute protein into solution. The samples were separated by SDS-PAGE and analyzed by western blotting.

### Protein half-life assay

Each dish was added with cycloheximide treatment maintained for 0, 30, 60, 90, and 120 min separately at final concentration of 100 μg/ml. Then we collected and lysed cells, analyzed the relevant protein expression by western blotting utilizing anti-HBP1 antibody. Quantification of expression of HBP1 protein under different time points was determined utilizing Image J software and normalized to GAPDH.

### In vivo ubiquitination

293T cells were co-transfected with various plasmids. 18 h after transfection, cells were added with MG132 (Merck Millipore) at final concentration of 10 μM lasting for 6 h, and then we collected and lysed cells. The samples were incubated with anti-HA antibody in addition with protein A-Sepharose (GE Healthcare) and separated by SDS-PAGE and analyzed by western blotting.

### In vitro ubiquitination and mass spectrometry

We performed In vitro ubiquitination assay utilizing MDM2/p53 Ubiquitination Kit (Boston Biochem). The purified His-tagged HBP1 and E1 enzyme, E2 enzyme, GST-MDM2, ubiquitin, Mg2+-ATP were incubated with Reaction Buffer 1 h at 30 °C water bath, and the mixture were subsequently separated by SDS-PAGE and analyzed by western blotting or Coomassie blue staining for mass spectrometry.

### Bisulfite sequencing

The genome DNA was treated referring to the manufacturer’s instruction by EpiTect Bisulfite Kit (Qiagen). The primers’ sequences for p21 and p16 promoters were shown as follows: p21 promoter, 5′-GGGAGGAGGGAAGTGTTTT-3′ and 5′-ACAACTACTCACACCTCAACT-3′ (24 CpG islands), and p16 promoter, 5′-TAGGGGGATATTTTTTAG-3′ and 5′-TATCTTTCCAAACAA-3′ (28 CpG islands). The PCR products were purified form the gel (Tiangen), then the fragments were ligated into a pGEM-T vector utilizing the TA cloning system (Tiangen). For analysis of methylation rate, we chose at least 10 clones randomly to perform sequencing analysis.

### Immunocytochemistry

Cells were seeded into 3.5 cm confocal dishes. After transfection or ionizing radiation treatment, cells were fixed with 4% paraformaldehyde for 15 min and permeabilized (using 0.2% Triton X-100 in PBS) for 15 min under room temperature. To detect 5-methylcytosine, cells were treated with 2 M HCl for extra 30 min. After sufficient washing with PBS, cells were blocked for 1 h under room temperature (using 1% bovine serum albumin in PBS). Cells were incubated with anti-γ-H2AX antibody or anti-5-methylcytosine antibody, followed by secondary antibodies labeled with AlexaFlour546 (anti-mouse IgG) or Cy3 (anti-rabbit IgG). DNA was stained with DAPI at final concentration of 1 μg/ml. Images were photographed utilizing confocal microscope (Olympus).

### MTT assay

Stable transfected cell sublines were constructed by lentivirus infection. Cells were seeded at a density of 1000 cells per well into 96-well plates. After culturing for 1–7 days separately, 15 μl of MTT solution (5 mg/ml) was added to each well, and incubated at 37 °C lasting for 4 h. Then we removed the medium and added 200 μl DMSO to each well to dissolve the formazan crystals. The samples were detected under absorbance peak at 490 nm utilizing the microplate reader.

### EdU incorporation assay

To perform EdU incorporation assay we used the EdU incorporation assay kit (Ribobio) and followed the manufacturer’s protocol. All cells were detected utilizing fluorescence microscopy (Leica). We counted at least 300 cells among randomly chosen fields for statistical analysis.

### Soft agar colony formation assay

Stable transfected cell sublines were constructed by lentivirus infection. About 3 × 10^4^ cells were seeded in 6-well plate with 2 ml of DMEM containing 10% FBS and 0.35% agar on a layer of 2 ml of the same medium containing 0.7% agar. Two weeks after culture, we observed the cell samples and took photographs and counted colonies.

### Tumorigenicity in nude mice

Stable transfected cell sublines were constructed by lentivirus infection. About 2 × 10^6^ cells were suspended in 200 μl of PBS. Six-week-old male nude mice were subcutaneously injected with these cells into left or right hind legs. After injection for 4 weeks, the mice were killed, and the tumors were weighed and measured. All experiments and facilities were approved by the Committee for Ethics of Animal Experiments and were conducted in conformity to the Guidelines for Animal Experiments, Peking University Health Science Center.

### Statistical analysis

Statistical analyses were performed using SPSS 17.0 software. The data are expressed as the mean ± S.D. (standard error of mean) from at least three-independent experiments. The data in statistical tests conformed to normal distribution and variance are similar. The statistical analysis was performed by using Student’s *t*-test, and *p* < 0.01 or 0.05 was considered significant. *p < 0.05, **p < 0.01.

## Supplementary information

supplementary figure legends.

figure S1

figure S2

figure S2

## References

[1] Fakharzadeh SS, Trusko SP, George DL (1991). Tumorigenic potential associated with enhanced expression of a gene that is amplified in a mouse tumor cell line. EMBO J.

[2] Finlay CA (1993). The mdm-2 oncogene can overcome wild-type p53 suppression of transformed cell growth. Mol Cell Biol.

[3] Eischen CM, Weber JD, Roussel MF, Sherr CJ, Cleveland JL (1999). Disruption of the ARF-Mdm2-p53 tumor suppressor pathway in Myc-induced lymphomagenesis. Genes Dev.

[4] Momand J, Jung D, Wilczynski S, Niland J (1998). The MDM2 gene amplification database. Nucleic Acids Res.

[5] Rayburn E, Zhang R, He J, Wang H (2005). MDM2 and human malignancies: expression, clinical pathology, prognostic markers, and implications for chemotherapy. Curr Cancer Drug Targets.

[6] Bond GL, Hu W, Bond EE, Robins H, Lutzker SG, Arva NC (2004). A single nucleotide polymorphism in the MDM2 promoter attenuates the p53 tumor suppressor pathway and accelerates tumor formation in humans. Cell.

[7] Mendrysa SM, O’Leary KA, McElwee MK, Michalowski J, Eisenman RN, Powell DA (2006). Tumor suppression and normal aging in mice with constitutively high p53 activity. Genes Dev.

[8] Wang P, Lushnikova T, Odvody J, Greiner TC, Jones SN, Eischen CM (2008). Elevated Mdm2 expression induces chromosomal instability and confers a survival and growth advantage to B cells. Oncogene.

[9] Ganguli G, Wasylyk B (2003). p53-independent functions of MDM2. Mol Cancer Res.

[10] Jones SN, Hancock AR, Vogel H, Donehower LA, Bradley A (1998). Overexpression of Mdm2 in mice reveals a p53-independent role for Mdm2 in tumorigenesis. Proc Natl Acad Sci USA.

[11] Bartel F, Taubert H, Harris LC (2002). Alternative and aberrant splicing of MDM2 mRNA in human cancer. Cancer Cell.

[12] Cordon-Cardo C, Latres E, Drobnjak M, Oliva MR, Pollack D, Woodruff JM (1994). Molecular abnormalities of mdm2 and p53 genes in adult soft tissue sarcomas. Cancer Res.

[13] Watanabe T, Ichikawa A, Saito H, Hotta T (1996). Overexpression of the MDM2 oncogene in leukemia and lymphoma. Leuk Lymphoma.

[14] Pan Y, Chen J (2003). MDM2 promotes ubiquitination and degradation of MDMX. Mol Cell Biol.

[15] Legube G, Linares LK, Lemercier C, Scheffner M, Khochbin S, Trouche D (2002). Tip60 is targeted to proteasome-mediated degradation by Mdm2 and accumulates after UV irradiation. EMBO J.

[16] Lin HK, Wang L, Hu YC, Altuwaijri S, Chang C (2002). Phosphorylation-dependent ubiquitylation and degradation of androgen receptor by Akt require Mdm2 E3 ligase. EMBO J.

[17] Yang JY, Zong CS, Xia W, Yamaguchi H, Ding Q, Xie X (2008). ERK promotes tumorigenesis by inhibiting FOXO3a via MDM2- mediated degradation. Nat Cell Biol.

[18] Yang JY, Zong CS, Xia W, Wei Y, Ali-Seyed M, Li Z (2006). MDM2 promotes cell motility and invasiveness by regulating E-cadherin degradation. Mol Cell Biol.

[19] Wang SP, Wang WL, Chang YL, Wu CT, Chao YC, Kao SH (2009). p53 controls cancer cell invasion by inducing the MDM2-mediated degradation of Slug. Nat Cell Biol.

[20] Yu J, Guo M, Wang T, Li X, Wang D, Wang X (2016). Inhibition of cell proliferation, migration and invasion by a glioma-targeted fusion protein combining the p53 C terminus and MDM2-binding domain. Cell Prolif.

[21] Iwasa H, Kudo T, Maimaiti S, Ikeda M, Maruyama J, Nakagawa K (2013). The RASSF6 tumor suppressor protein regulates apoptosis and the cell cycle via MDM2 protein and p53 protein. J Biol Chem.

[22] Wen W, Peng C, Kim MO, Ho Jeong C, Zhu F, Yao K (2014). Knockdown of RNF2 induces apoptosis by regulating MDM2 and p53 stability. Oncogene.

[23] Riley MF, You MJ, Multani AS, Lozano G (2016). Mdm2 overexpression and p73 loss exacerbate genomic instability and dampen apoptosis, resulting in B-cell lymphoma. Oncogene.

[24] Lundgren K, Montes de Oca Luna R, McNeill YB, Emerick EP, Spencer B, Barfield CR (1997). Targeted expression of MDM2 uncouples S phase from mitosis and inhibits mammary gland development independent of p53. Genes Dev.

[25] Alt JR, Bouska A, Fernandez MR, Cerny RL, Xiao H, Eischen CM (2005). Mdm2 binds to Nbs1 at sites of DNA damage and regulates double strand break repair. J Biol Chem.

[26] Wienken M, Dickmanns A, Nemajerova A, Kramer D, Najafova Z, Weiss M (2016). MDM2 associates with polycomb repressor complex 2 and enhances stemness-promoting chromatin modifications independent of p53. Mol Cell.

[27] Berasi SP, Xiu M, Yee AS, Paulson KE (2004). HBP1 repression of the p47phox gene: cell cycle regulation via the NADPH oxidase. Mol Cell Biol.

[28] Zhang X, Kim J, Ruthazer R, McDevitt MA, Wazer DE, Paulson KE (2006). The HBP1 transcriptional repressor participates in RAS-induced premature senescence. Mol Cell Biol.

[29] Pan K, Chen Y, Roth M, Wang W, Wang S, Yee AS (2013). HBP1-mediated transcriptional regulation of DNA methyltransferase 1 and its impact on cell senescence. Mol Cell Biol.

[30] Chen Y, Pan K, Wang P, Cao Z, Wang W, Wang S (2016). HBP1-mediated regulation of p21 protein through the Mdm2/p53 and TCF4/EZH2 pathways and its impact on cell senescence and tumorigenesis. J Biol Chem.

[31] Escamilla-Powers JR, Daniel CJ, Farrell A, Taylor K, Zhang X, Byers S (2010). The tumor suppressor protein HBP1 is a novel c-myc-binding protein that negatively regulates c-myc transcriptional activity. J Biol Chem.

[32] Li H, Wang W, Liu X, Paulson KE, Yee AS, Zhang X (2010). Transcriptional factor HBP1 targets P16 (INK4A), upregulating its expression and consequently is involved in Ras-induced premature senescence. Oncogene.

[33] Gartel AL, Goufman E, Tevosian SG, Shih H, Yee AS, Tyner AL (1998). Activation and repression of p21 (WAF1/CIP1) transcription by RB binding proteins. Oncogene.

[34] Yao CJ, Works K, Romagnoli PA, Austin GE (2005). Effects of overexpression of HBP1 upon growth and differentiation of leukemic myeloid cells. Leukemia.

[35] Lemercier C, Duncliffe K, Boibessot I, Zhang H, Verdel A, Angelov D (2000). Involvement of retinoblastoma protein and HBP1 in histone H10 gene expression. Mol Cell Biol.

[36] Saadatzadeh MR, Elmi AN, Pandya PH, Bijangi-Vishehsaraei K, Ding J, Stamatkin CW (2017). The role of MDM2 in promoting genome stability versus instability. Int J Mol Sci.

[37] Deb SP, Singh S, Deb S (2014). MDM2 overexpression, activation of signaling networks, and cell proliferation. Subcell Biochem.

[38] Eischen CM (2017). Role of Mdm2 and Mdmx in DNA repair. J Mol Cell Biol.

[39] Wang P, Greiner TC, Lushnikova T, Eischen CM (2006). Decreased Mdm2 expression inhibits tumor development induced by loss of ARF. Oncogene.

[40] Carroll PE, Okuda M, Horn HF, Biddinger P, Stambrook PJ, Gleich LL (1999). Centrosome hyperamplification in human cancer: chromosome instability induced by p53 mutation and/or Mdm2 overexpression. Oncogene.

[41] Li Q, Lozano G (2013). Molecular pathways: targeting Mdm2 and Mdm4 in cancer therapy. Clin Cancer Res.

[42] Xiu M, Kim J, Sampson E, Huang CY, Davis RJ, Paulson KE (2003). The transcriptional repressor HBP1 is a target of the p38 mitogen-activated protein kinase pathway in cell cycle regulation. Mol Cell Biol.

[43] Wang S, Cao Z, Xue J, Li H, Jiang W, Cheng Y (2017). A positive feedback loop between Pim-1 kinase and HBP1 transcription factor contributes to hydrogen peroxide-induced premature senescence and apoptosis. J Biol Chem.

[44] Ciccia A, Elledge SJ (2010). The DNA damage response: making it safe to play with knives. Mol Cell.

[45] Meulmeester E, Maurice MM, Boutell C, Teunisse AF, Ovaa H, Abraham TE (2005). Loss of HAUSP-mediated deubiquitination contributes to DNA damage-induced destabilization of Hdmx and Hdm2. Mol Cell.

[46] Stommel JM, Wahl GM (2004). Accelerated MDM2 auto-degradation induced by DNA-damage kinases is required for p53 activation. EMBO J.

[47] Jazayeri A, Falck J, Lukas C, Bartek J, Smith GC, Lukas J (2006). ATM- and cell cycle-dependent regulation of ATR in response to DNA double-strand breaks. Nat Cell Biol.

[48] Slack A, Chen Z, Tonelli R, Pule M, Hunt L, Pession A (2005). The p53 regulatory gene MDM2 is a direct transcriptional target of MYCN in neuroblastoma. Proc Natl Acad Sci USA.

[49] Li Y, Choi PS, Casey SC, Dill DL, Felsher DW (2014). MYC through miR-17-92 suppresses specific target genes to maintain survival, autonomous proliferation, and a neoplastic state. Cancer Cell.

[50] Chène P (2004). Inhibition of the p53-MDM2 interaction: targeting a protein-protein interface. Mol Cancer Res.

[51] Zhao Y, Aguilar A, Bernard D, Wang S (2015). Small-molecule inhibitors of the MDM2-p53 protein-protein interaction (MDM2 Inhibitors) in clinical trials for cancer treatment. J Med Chem.

[52] Lehmann C, Friess T, Birzele F, Kiialainen A, Dangl M (2016). Superior anti-tumor activity of the MDM2 antagonist idasanutlin and the Bcl-2 inhibitor venetoclax in p53 wild-type acute myeloid leukemia models. J Hematol Oncol.

[53] Zhou Y, Perez RE, Duan L, Maki CG (2018). DZNep represses Bcl-2 expression and modulates apoptosis sensitivity in response to Nutlin-3a. Cancer Biol Ther.

