# Supplementary material for: MDM2 promotes genome instability by ubiquitinating the transcription factor HBP1
Source: Oncogene. 2019 Feb 28;38(24):4835–55. doi: 10.1038/s41388-019-0761-2 (PMC6756050; doi:10.1038/s41388-019-0761-2)
Supplement: Supplementary file 1 — supplementary figure legends. [file 41388_2019_761_MOESM1_ESM.doc]

**Fig. S1** Results of mass spectrometry showed that K144 and K398 of HBP1 were ubiquitinated in the presence of MDM2.

**Fig. S2** HBP1 alters methylation and tumorigenesis in MDM2 overexpressing cell lines. (a)HBP1 overexpression decreases the protein levels of DNMT1, EZH2 and H3K27me3. MCF-7 and MDA-MB-231 cells were transfected with HBP1 or control vector. The protein levels of HBP1, DNMT1, EZH2, H3K27me3, and histone H3 were measured by western blotting. Level of GAPDH was used as a loading control. (b) HBP1 overexpression decreases global DNA hypermethylation. Cells described in Fig. S2a were stained with antibodies specific for the 5mC epitope (red). (c) and (d) HBP1 overexpression decreases cell growth. EdU incorporation (c) and MTT (d) assays were conducted with MCF-7 and MDA-MB-231 cells stably transfected with HBP1 or control vector. The mean ± S.D. for three independent experiments are shown. *, *p* < 0.05. (e) HBP1 overexpression decreases cell growth in soft agar. Soft agar colony formation assay of the cells described in Fig. S2c. Cells were cultured in soft agar for 2 weeks (top panel). The colony numbers in three different microscope fields were counted and are shown as mean ± S.D. (bottom panel) **, *p* < 0.01. (f) HBP1 overexpression decreases tumorigenesis. Cells described in Fig. S2c were subcutaneously injected into nude mice. Four weeks after injection, the tumors were weighed, and size was measured. Data are shown as mean ± S.D. (n=3). **, *p* < 0.01.
