# Supplementary figures and images for: MDM2 promotes genome instability by ubiquitinating the transcription factor HBP1
Source: Oncogene. 2019 Feb 28;38(24):4835–55. doi: 10.1038/s41388-019-0761-2 (PMC6756050; doi:10.1038/s41388-019-0761-2)

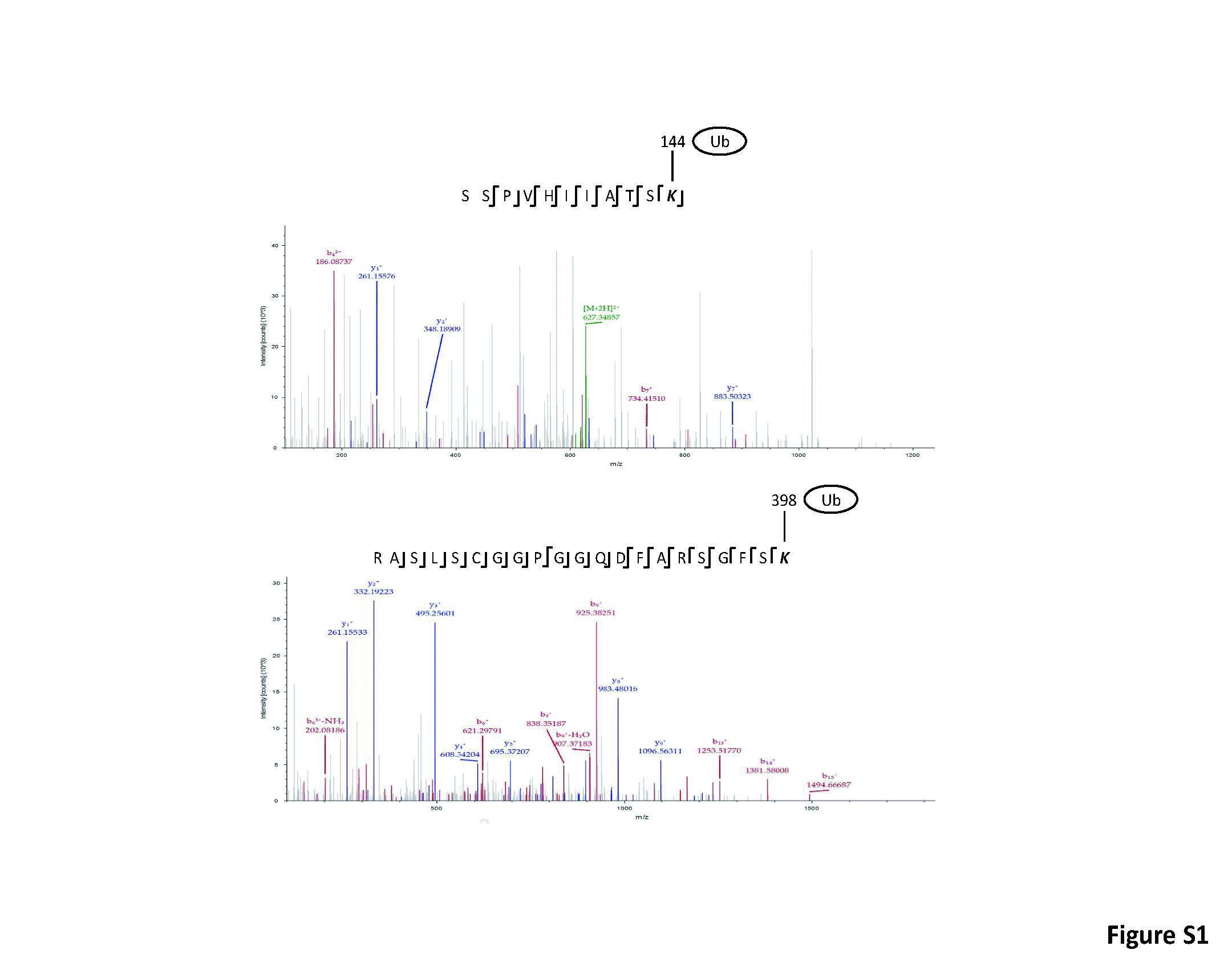

Supplement: Supplementary file 2 — figure S1 [file 41388_2019_761_MOESM2_ESM.jpg]

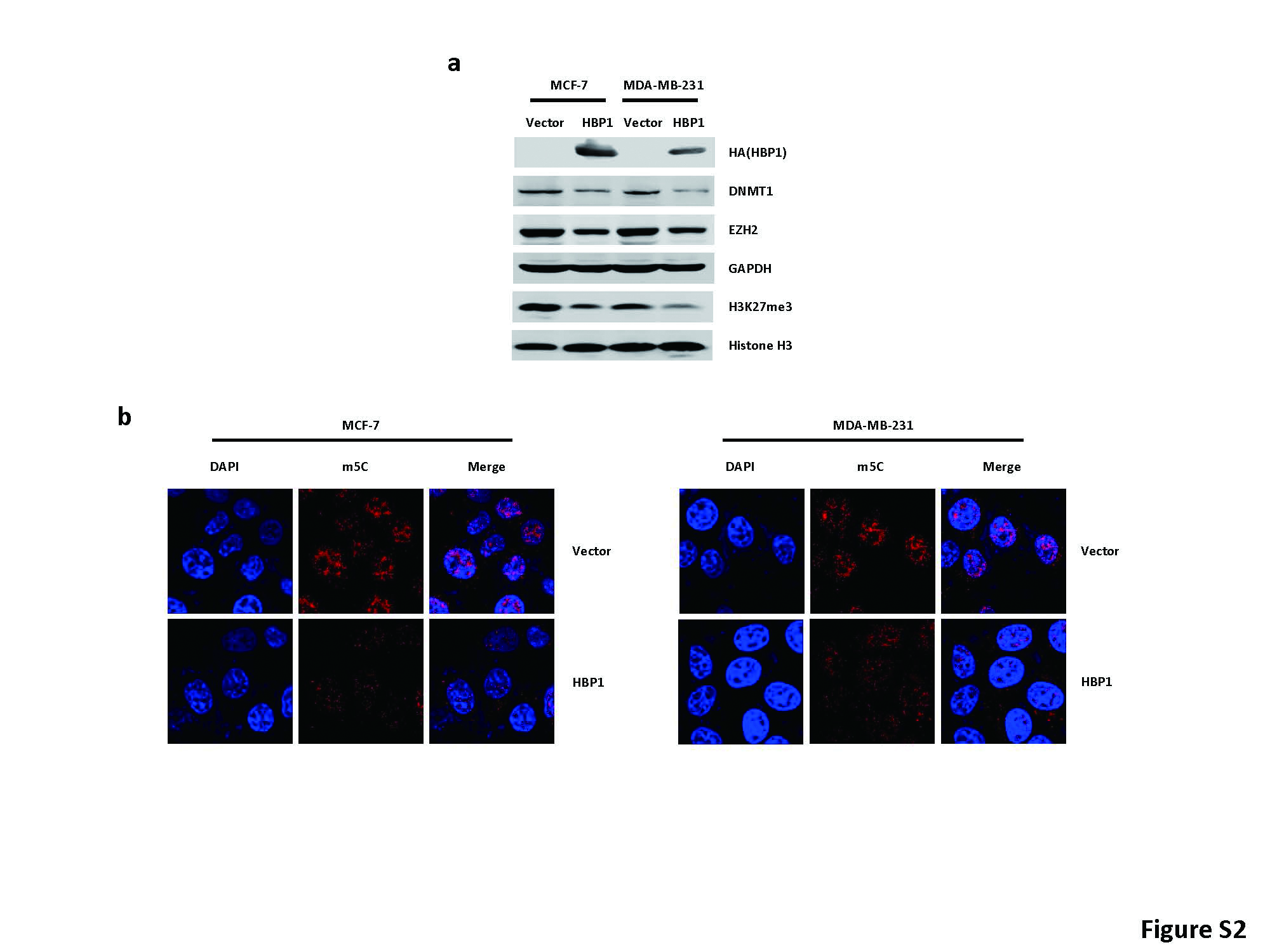

Supplement: Supplementary file 3 — figure S2 [file 41388_2019_761_MOESM3_ESM.jpg]

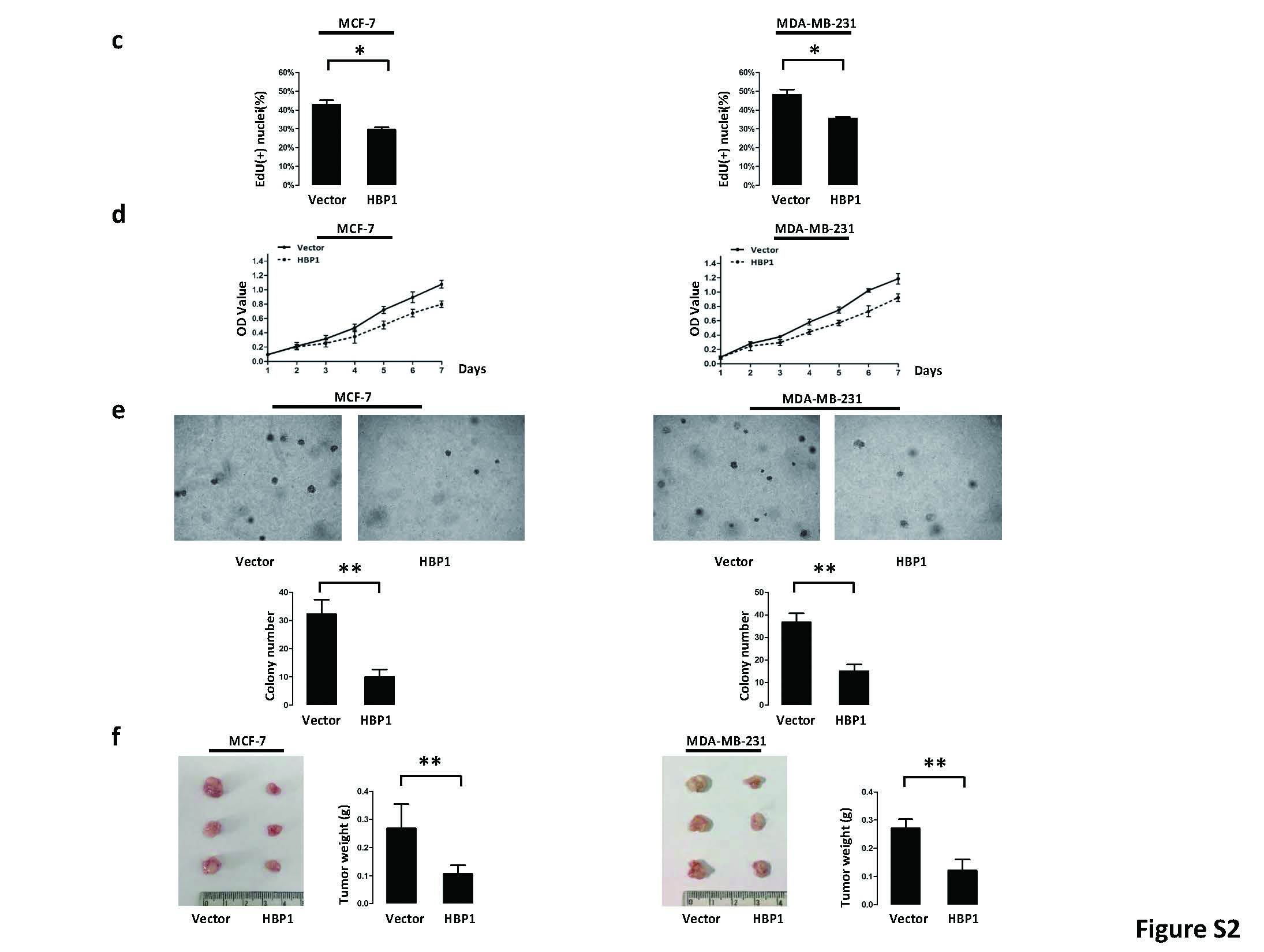

Supplement: Supplementary file 4 — figure S2 [file 41388_2019_761_MOESM4_ESM.jpg]
